# The 2018 correlative microscopy techniques roadmap

**DOI:** 10.1088/1361-6463/aad055

**Published:** 2018-08-31

**Authors:** Toshio Ando, Satya Prathyusha Bhamidimarri, Niklas Brending, H Colin-York, Lucy Collinson, Niels De Jonge, P J de Pablo, Elke Debroye, Christian Eggeling, Christian Franck, Marco Fritzsche, Hans Gerritsen, Ben N G Giepmans, Kay Grunewald, Johan Hofkens, Jacob P Hoogenboom, Kris P F Janssen, Rainer Kaufmann, Judith Klumperman, Nyoman Kurniawan, Jana Kusch, Nalan Liv, Viha Parekh, Diana B Peckys, Florian Rehfeldt, David C Reutens, Maarten B J Roeffaers, Tim Salditt, Iwan A T Schaap, Ulrich S Schwarz, Paul Verkade, Michael W Vogel, Richard Wagner, Mathias Winterhalter, Haifeng Yuan, Giovanni Zifarelli

**Affiliations:** 1 Nano Life Science Institute (WPI-NanoLSI), Kanazawa University, Kanazawa, Japan; 2 Department of Life Sciences & Chemistry, Jacobs University, Bremen, Germany; 3 Ionovation GmbH, Osnabrück, Germany; 4 MRC Human Immunology Unit, Weatherall Institute of Molecular Medicine, University of Oxford, Headley Way, OX3 9DS Oxford, United Kingdom; 5 Francis Crick Institute, London, United Kingdom; 6 INM—Leibniz Institute for New Materials, 66123 Saarbrücken, Germany; 7 Saarland University, 66123 Saarbrücken, Germany; 8 Dpto. Física de la Materia Condensada Universidad Autónoma de Madrid 28049, Madrid, Spain; 9 Instituto de Física de la Materia Condensada IFIMAC, Universidad Autónoma de Madrid 28049, Madrid, Spain; 10 KU Leuven, Department of Chemistry, B-3001 Heverlee, Belgium; 11 Institute of Applied Optics, Friedrich-Schiller University, Jena, Germany; 12 Leibniz Institute of Photonic Technology (IPHT), Jena, Germany; 13 Department of Mechanical Engineering, University of Wisconsin-Madison, 1513 University Ave, Madison, WI 53706, United States of America; 14 Kennedy Institute for Rheumatology, University of Oxford, Oxford, United Kingdom; 15 Debye Institute, Utrecht University, Utrecht, Netherlands; 16 Department of Cell Biology, University of Groningen, University Medical Center Groningen, Groningen, Netherlands; 17 Division of Structural Biology, Wellcome Trust Centre for Human Genetics, University of Oxford, Oxford, United Kingdom; 18 Centre of Structural Systems Biology Hamburg and University of Hamburg, Hamburg, Germany; 19 Heinrich-Pette-Institute, Leibniz Institute of Virology, Hamburg, Germany; 20 Imaging Physics, Delft University of Technology, Delft, Netherlands; 21 Department of Biochemistry, University of Oxford, Oxford, United Kingdom; 22 Section Cell Biology, Center for Molecular Medicine, University Medical Center Utrecht, Utrecht University, Heidelberglaan 100, 3584CX Utrecht, Netherlands; 23 Centre for Advanced Imaging, The University of Queensland, Brisbane, QLD 4072, Australia; 24 University Hospital Jena, Jena, Germany; 25 Faculty of Medicine, Saarland University, 66421 Homburg, Germany; 26 University of Göttingen, Third Institute of Physics—Biophysics, 37077 Göttingen, Germany; 27 KU Leuven, Department of Bioscience Engineering, B-3001 Heverlee, Belgium; 28 University of Göttingen, Institute for X-Ray Physics, 37077 Göttingen, Germany; 29 SmarAct GmbH, Schütte-Lanz-Str. 9, D-26135 Oldenburg, Germany; 30 Institute for Theoretical Physics and BioQuant, Heidelberg University, Heidelberg, Germany; 31 School of Biochemistry, University of Bristol, Bristol, United Kingdom; 32 Department of Physiology, Anatomy and Genetics, University of Oxford, Oxford, United Kingdom; christian.eggeling@rdm.ox.ac.uk; J.P.Hoogenboom@tudelft.nl; Schaap@smaract.com

**Keywords:** correlative microscopy, fluorescence microscopy, x-ray microscopy, electron microscopy, magnetic resonance imaging, atomic force microscopy, super-resolution microscopy

## Abstract

Developments in microscopy have been instrumental to progress in the life sciences, and many new techniques have been introduced and led to new discoveries throughout the last century. A wide and diverse range of methodologies is now available, including electron microscopy, atomic force microscopy, magnetic resonance imaging, small-angle x-ray scattering and multiple super-resolution fluorescence techniques, and each of these methods provides valuable read-outs to meet the demands set by the samples under study. Yet, the investigation of cell development requires a multi-parametric approach to address both the structure and spatio-temporal organization of organelles, and also the transduction of chemical signals and forces involved in cell–cell interactions. Although the microscopy technologies for observing each of these characteristics are well developed, none of them can offer read-out of all characteristics simultaneously, which limits the information content of a measurement. For example, while electron microscopy is able to disclose the structural layout of cells and the macromolecular arrangement of proteins, it cannot directly follow dynamics in living cells. The latter can be achieved with fluorescence microscopy which, however, requires labelling and lacks spatial resolution. A remedy is to combine and correlate different readouts from the same specimen, which opens new avenues to understand structure–function relations in biomedical research. At the same time, such correlative approaches pose new challenges concerning sample preparation, instrument stability, region of interest retrieval, and data analysis. Because the field of correlative microscopy is relatively young, the capabilities of the various approaches have yet to be fully explored, and uncertainties remain when considering the best choice of strategy and workflow for the correlative experiment. With this in mind, the Journal of Physics D: Applied Physics presents a special roadmap on the correlative microscopy techniques, giving a comprehensive overview from various leading scientists in this field, via a collection of multiple short viewpoints.

**List of abbreviations**
CLEM: Correlative light and electron microscopy
TEM: Transmission electron microscope
SEM: Scanning electron microscope
FIB: Focussed ion beam
CL: Cathodoluminescence
EDX: Energy-dispersive-x-ray analysis
EELS: Electron energy loss spectroscopy
nano-SIMS: Secondary ion mass spectroscopy at the nanoscale
STEM: Scanning transmission electron microscopy
ESEM: Environmental scanning electron microscopy
EMPIAR: Electron microscopy public image archive
LM: Light microscopy
FRET: Förster resonance energy transfer
STORM: Stochastic optical reconstruction microscopy
PALM: Photoactivated localization microscopy
AFM: Atomic force microscopy
FM: Fluorescence microscopy
GFP: Green fluorescent protein
FP: Fluorescent protein
NA: Numerical aperture
SiN: Silicon nitride
QD: Quantum dot
NSOM: Near-field scanning optical microscopy
PFS: Point spread function
FCS: Fluorescence correlation spectroscopy
PMT: Photomultiplier
MRI: Magnetic resonance imaging
fMRI: Functional magnetic resonance imaging
dfMRI: Diffusion functional magnetic resonance imaging
NMR: Nuclear magnetic resonance
SAXS: Small angle x-ray scattering
ROI: Region of interest
2D: two dimensional
3D: three dimensional
PIV: Particle image velocimetry
SPT: Single particle tracking
PCA: Principal component analysis
TFM: Traction force microscopy
STFM: Super resolution traction force microscopy
HLB: Horizontally oriented bilayer
OSTR: Optical single transporter recording
VCF: Voltage-clamp fluorometry
PCF: Patch-clamp fluorometry
BOLD: Blood oxygenation-dependent

## Integrated light and electron microscopy

### Hans C Gerritsen^1^ and Jacob P Hoogenboom^2^

^1^ Debye Institute, Utrecht University, Utrecht, Netherlands

^2^ Imaging Physics, Delft University of Technology, Delft, Netherlands

#### Status.

The integration of a light microscope (LM) and an electron microscope (EM) into a single apparatus has been pursued for doing correlative light and electron microscopy (CLEM) experiments since the 1980s. Technological advances and the renewed interest for CLEM have led to several novel, improved systems, both for transmission EM (TEM) and scanning EM (SEM) (see overviews [1, 2]). Integrated CLEM approaches improve correlation or image registration accuracy, facilitate the retrieval of (rare) regions of interest, reduce CLEM operation times, and/or avoid sample contamination in (cryo-) transfer [3]. Potential drawbacks for integrated microscopy are the need for fluorescence preservation during preparation for EM and in vacuum, and auto-fluorescence of some resin materials. Preparation schemes using reduced concentrations of osmium and metal salts [4–6] as well as osmium-resistant genetic labels [7] have evolved, while extension to integrated cryo-microscopy [8, 9] may alleviate all these issues.

**Figure 1.**
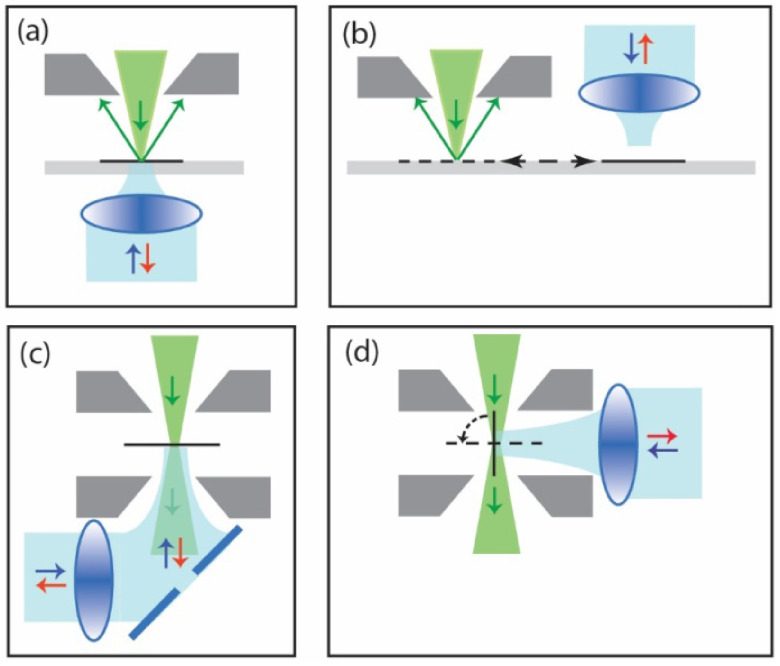
Schematic indication of realizations for integrated LM inside ((a), (b)) scanning or ((c), (d)) transmission EMs. Designs can be distinguished based on whether ((a), (c)) both microscopes share the same field of view, or (b) a translation, or (d) rotation is needed, to switch from light to electron microscopy and vice versa. Electron beam is indicated in green, light beam in blue.

**Figure 2.**
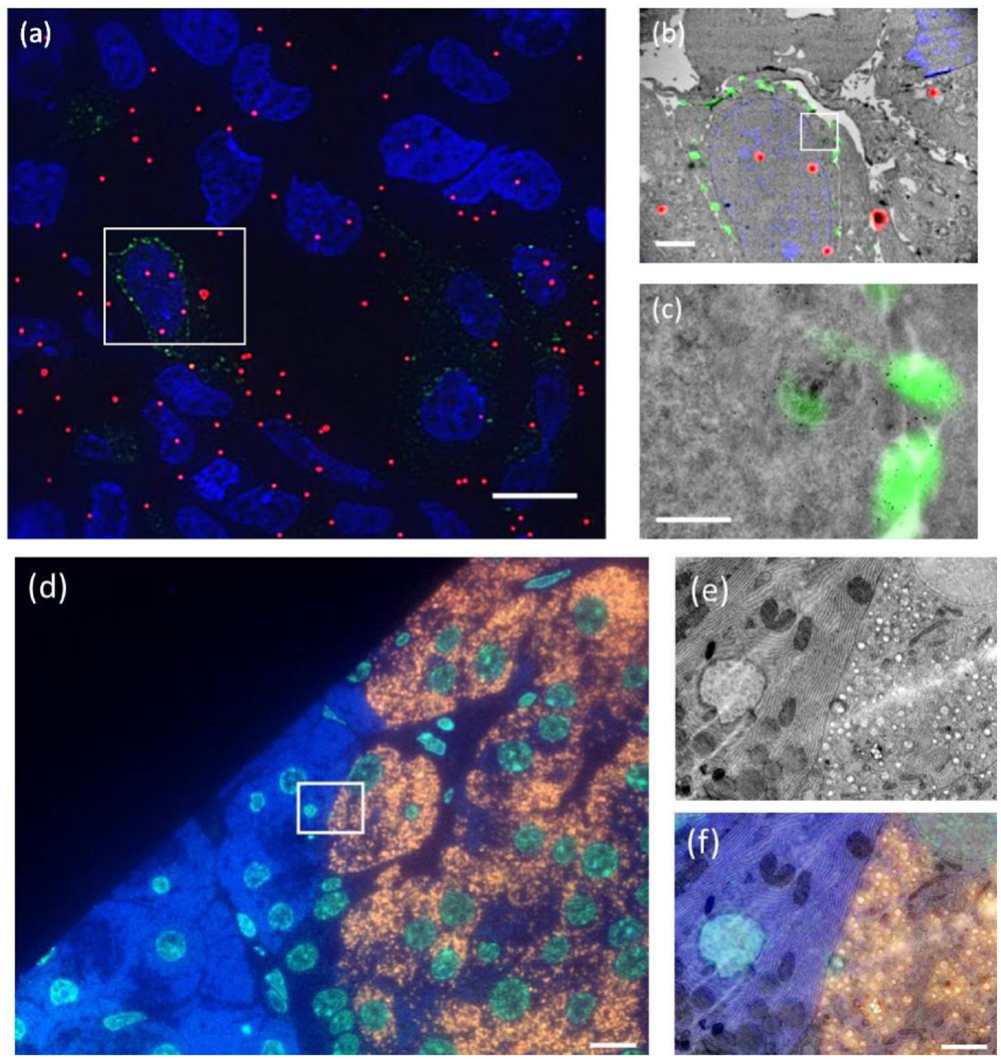
Examples of (a)–(c) fiducial and (d)–(f) non-fiducial based image registration in integrated microscopes. (a) FM image in TEM (implementation according to figure 1(d)) of Tokuyasu sections of HeLa cells transfected with LAMP-1-GFP. Nuclei are shown in blue (DAPI), LAMP-1-GFP in green and fiducials in red. (b) Overlay of ROI (boxed area in (a)) of fluorescence and TEM images. (c) Zoom in on LAMP-1-GFP rich area. Fiducials consist of silica particles with a 15 nm gold core and a 40 nm fluorescently labeled silica shell. Overlay accuracy is about 30 nm. (d) FM image in SEM (implementation according to figure 1(a)) of rat pancreas sections, immuno-labelled after embedding in epon to show nuclei in blue (Hoechst), guanine quadruplexes in light blue (Alexa488), and insulin in orange (Alexa594). (e) SEM image of the ROI (boxed area in (d)). (f) Overlay of fluorescence from the ROI with the SEM image. The overlay (<20 nm accuracy) is obtained via an automated registration procedure between both microscopes [10]. Scale bars are 10 *µ*m in (a), (d), 2 *µ*m in (b), (e) and (f), and 0.5 *µ*m in (c).

Several types of integrated microscopes are now commercially available and increasingly finding their way into the laboratory environment. These can be roughly divided in two variants: systems where EM and integrated LM share the same field of view (figures 1(a) and (c)), and those where the sample needs to be translated or rotated within the vacuum chamber in between imaging with both modalities (figures 1(b) and (d) [2]. In the first variant, image registration does not need fiducial markers (see figure 2), but can be done using so-called cathodoluminescence pointers, which can be extremely accurate (<10 nm) and automated [10], but sample thickness is limited by the depth of view of the LM. The latter case is applicable to (cryo-)TEM and can be used in SEMs, removing the restriction on sample thickness. Either case may hold considerable benefits to target key challenges in EM and CLEM. Larger samples are used in volume-EM where the sample is trimmed with focused ion beams (FIB) or *in situ* microtomes [11, 12]. Automated and highly accurate integrated CLEM may be key for superresolution (SR) fluorescence localization of bio-molecules in EM images [13], for locating and trimming sections for sub-nm resolution structural cryo-EM [12], and for large-scale serial section EM [11]. A recent demonstration of integrated SR fluorescence CLEM showed a localization accuracy of 50 nm [14], comparable to routine stand-alone SR experiments.

### Current and future challenges.

#### SR CLEM.

Combining SR fluorescence with EM holds the promise of precisely pinpointing molecules that cannot be labelled for EM in EM images. SR CLEM opens the door to functional imaging of, for instance, specific lipids, ions or enzymatic activity in the ultrastructural image obtained with EM. Ultimately, localization accuracy should be comparable to the nanometer resolution of EM, effectively adding fluorescence contrast to EM. Current localization based SR techniques, such as photoactivated localization microscopy and stochastic optical reconstruction microscopy, routinely obtain resolutions of 20–50 nm. However, below 10 nm resolution, SR-EM registration accuracy and/or distortions induced by sample preparation become dominant. At this length scale, integrated microscopes and optimized ‘integrated’ specimen preparations are likely to yield the best results, because distortions due to specimen handling can be avoided and registration accuracy is high.

#### Cryo-CLEM.

Revolutionary developments in cryo-EM and electron tomography (ET) resulted in near-atomic resolution that enables resolving the internal structure of proteins. A major advantage of EM over crystallography approaches would be imaging the structure of a protein in its native, cryo-fixed environment. The holy grail in cryo-EM is to pinpoint a protein of interest in a cryo-fixed specimen and cut out a sufficiently thin slice (100–200 nm) containing this protein for transfer to cryo-EM/ET. FIB SEMs are the tool of choice for slicing, and cryo-fluorescence microscopy can highlight the protein of interest. A major challenge is to reach the precision needed for targeted 100–200 nm slicing in cryo-fixed cells, which can most probably only be reached with cryo-integrated fluorescence FIB-SEM. Challenges include accurate 3D correlation, especially considering the poor depth resolution in (confocal) microscopes and optical distortions.

#### Volume- and high-throughput EM.

The throughput in EM has evolved considerably. Large areas can be covered thanks to technical developments, e.g. increasing the size of sections cut by the microtome and using SEM for seamless imaging, as well as developments in software and automation, such as automated image acquisition and stitching. Volumes can be covered by an automated collection of thin serial sections and these imaging sections sequentially, or by imaging the upper face of the resin block followed by *in situ* trimming using an integrated microtome or FIB-SEM (see section 7). The recent acquisition of a zebrafish brain using serial-section SEM constitutes a hallmark example of what can be achieved with volume-EM [15]. However, data acquisition took over 200 full days of SEM operation, highlighting the need to pinpoint regions of interest to cut redundancy in acquisition, for which integrated CLEM seems excellently suited. Paired with the high-accuracy fluorescence-to-EM registration that can be obtained consistently over large areas, integrated microscopes seem particularly suited to improve throughput and functional mapping in serial sections volume-EM. Instrumentation seems to be in place, but automation, especially in fluorescence recognition and unattended acquisition, needs development. Challenges also remain in further, more wide-spread applications of fluorescence preserving EM sample preparation, on-section immuno-labelling, and reduction of resin auto-fluorescence. For block-face approaches, fiducial markers or calibration structures for 3D registration need further development.

### Advances in science and technology to meet challenges.

#### Fluorescence and photo-switching under EM conditions.

Optimized ‘integrated’ sample preparation is a common challenge to all of the approaches detailed above. For SR fluorescence, three hurdles need further attention. First, fluorescence has to survive fixation and other EM preparation steps, which has been achieved [1, 4–7], but needs a wider palette. Strong fixation and staining in 3D block-face requires the development of milder fixation procedures compatible with CLEM. Second, fluorescence and photo-switching has to be preserved in vacuum (dehydrated state); for most genetic fluorophores this results in low fluorescence quantum yield. Variable-pressure EM provides an alternative, but is time-consuming [14]. Techniques relying on direct electron beam excitation (cathodoluminescence) may provide alternatives (see section 2), but so far lack sufficiently small (<20 nm), stable probes. In addition, optimized genetic photo-switchable probes may provide a solution. Third, background fluorescence from resin may become problematic at low fluorescence signals (SR) or thick samples (3D CLEM). This requires developments of low auto-fluorescence resins and use of long wavelength fluorescent probes.

Much of these issues are alleviated with cryo-fixation, but photo-switching may be difficult, and illumination powers needed for SR microscopy may lead to local melting. Again, optimized fluorescent proteins for cryo-conditions are required, combined with the development of novel, cryo-specific SR techniques.

#### Registration and depth resolution in 3D.

While integrated microscopes allow for automatic image registration for thin 2D samples, registration of thicker 3D samples (volume CLEM) poses challenges. Novel fiducials are needed, which are bright enough in sample blocks, heavily stained with osmium and other metals and yield backscattered electron visibility in the stained blocks. Also, techniques to account for optical distortions (due to refractive index mismatch, for example) such as adaptive optics, need to be incorporated. Fluorescence localization in 3D requires optical sectioning, e.g. using an integrated confocal microscope. A major challenge is to achieve depth resolution matching that of volume-EM techniques, i.e.  ⩽100 nm.

#### Automation.

For 2D and 3D serial sections, instrumentation seems to be in place to move to a high throughput acquisition of large, accurately overlaid CLEM datasets [16]. Effort is required in the automation of data acquisition, drift and focus corrections and section recognition, all geared towards unattended, 24/7 operation. Further along the way, the automated recognition of target areas for EM, based on in-section fluorescence expression, may also help to enhance throughput in serial-section CLEM.

### Concluding remarks

A variety of integrated microscopes have emerged in recent years. Key application areas for integrated microscopy are in CLEM with super resolution fluorescence, CLEM for cryo- or volume-EM, and high-throughput CLEM based on serial sections. Sample preparation protocols and fluorescence probes for integrated CLEM are available, but in general, solutions require both further and broader optimization of genetic and organic fluorophores towards EM conditions (cryo, vacuum, use of osmium and other stains), methods for 3D registration and resolution matching, and development of automated data acquisition strategies. With these steps made, integrated microscopes may allow recording of precisely overlaid datasets of functional fluorescence and structural electron data crossing scales from the multi-cellular down to the molecular level.

### Acknowledgments

We thank our group members and collaborators for input and discussions. We acknowledge funding from Microscopy Valley, a research program supported by NWO-TTW Perspectief voor de Topsectoren (projects 12713, 12714 and 12715). J P H has a financial interest in Delmic BV, a company producing integrated microscopes.

## Multiscale multimodal multicolor microscopy

### Ben N G Giepmans

Department of Cell Biology, University of Groningen, University Medical Center Groningen, Groningen, Netherlands

#### Status.

Correlative light microscopy and electron microscopy (CLEM) is a key approach to studying structure–function relationships in cell biology. CLEM allows a biological process and building block (molecule, organelle, cell) to be identified and dynamically studied using fluorescent markers, followed by high-resolution analysis of the ultrastructural context with EM. In the past few decades, sample preparation steps, technical approaches, probes, microscopes and image analysis have been optimized to make CLEM a routine approach applied by many labs to date [1].

The strength of biomedical EM obviously lies in revealing the ultrastructure and detecting targets at a biomolecular scale, which is typically a few nanometers (nm) for proteins. In contrast, in fluorescence microscopy only the targets (typically restricted to three) are highlighted, with limited information on the non-labelled structures. The resolution and localization precision of fluorescence imaging has long been restricted to a sub-micrometer scale because of the diffraction limit. The rise of nanoscopy, i.e. light microscopy beyond the diffraction limit, directly showed a high potential for defining the localization of organelles with high precision (<0.1 *µ*m; see [1, 17]). Recently, Hell *et al* [17] developed ‘MINFLUX’, leading to nm-precision localization of labelled fluorescent molecules with a lateral resolution of 6 nm, enabling cell biology at the ultimate scale of biomolecules, but only for one or two targets at a time.

#### Current and future challenges.

**Figure 3.**
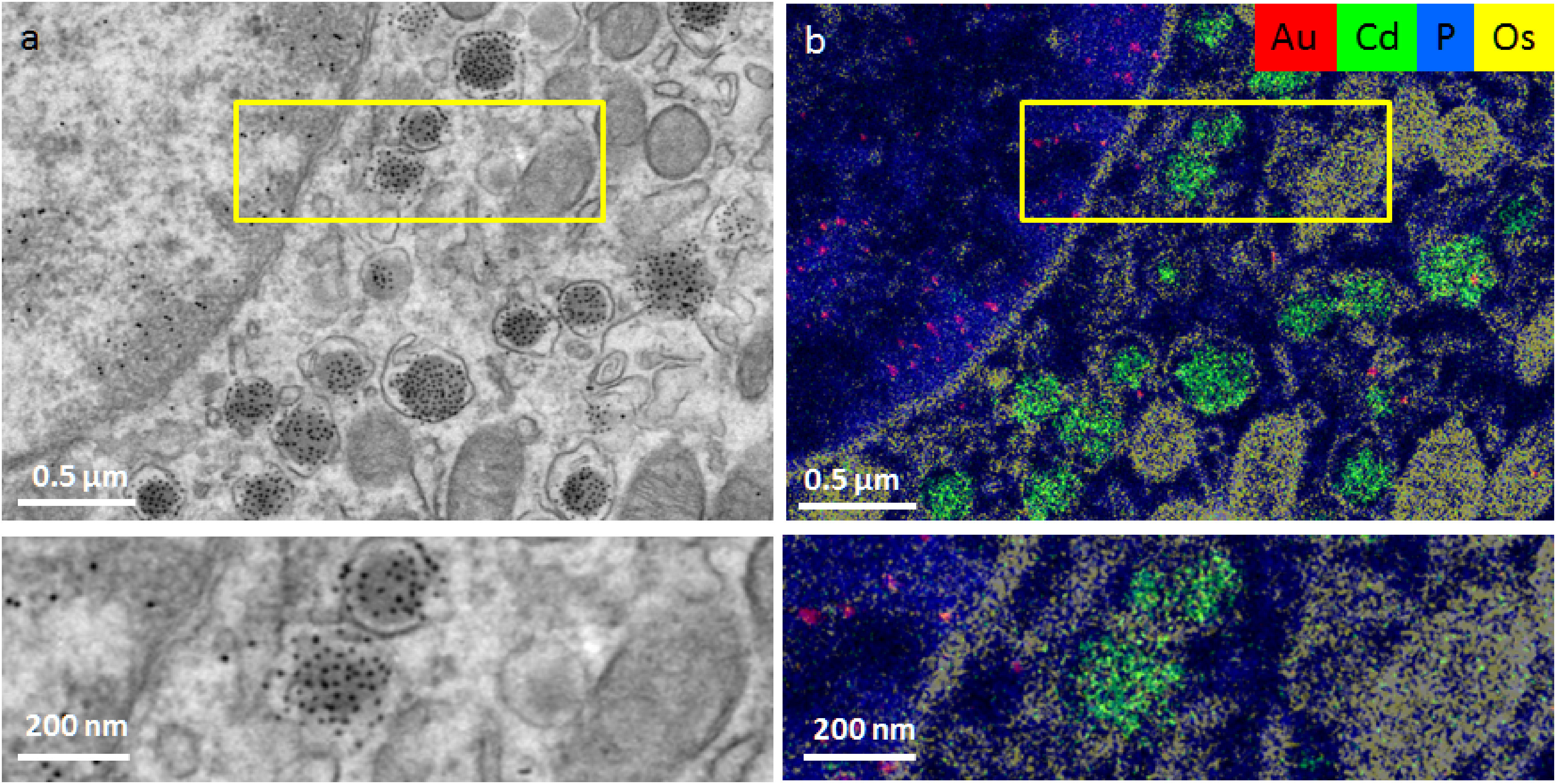
‘ColorEM’ using elemental analysis by energy dispersive x-ray imaging. ColorEM: label-free (P), paint (Os) and labeling DNA (Au) and peptides (Cd) is compatible. (a) Part of an islet of Langerhans immuno-labeled for structures in DNA (10 nm gold) and insulin (QD). (b) Overlay image of Au (red), Cd (green), Os (yellow) and P (blue) allows identification of G4 structures (gold labels) and insulin (Cd). Note the localization of Au to heterochromatin enriched in P, whereas the Cd signal is enclosed within a combination of Os rings and P that likely identifies phospholipid membranes of the vesicles. Large scale data and full resolution data is available via www.nanotomy.org; Reproduced from [22]. CC BY 4.0.

Although all microscopic pre-embedding labelling procedures often lead to the extraction of biomolecules, only EM painfully shows the effect on the ultrastructure [18]. Well-established ways to circumvent cellular damag, by introducing probes are by using either genetically-encoded tags and/or small molecules that do not need permeabilization [1] or post-embedding labelling, e.g. figure 3. The concession in CLEM for sample preparation, not optimal for either modality, also counts for the light microscopic analysis. Typically, fluorescence is poorly retained during EM sample preparation focussed on retaining optimal ultrastructure, although probes and procedures are being developed to retain fluorescence during embedding (reviewed in [1]), or fluorescent labels can be targeted post-embedding.

Another challenge in CLEM is to deal with large datasets. The successes of CLEM approaches have resulted in a demand to image large regions of interest (ROIs), typically easily performed with LM, at the typical resolution for EM (nm-scale). In dynamic vital microscopy followed by EM, this gap will not be easy to routinely solve, especially because of the difficulty of correlating data in the axial direction. In fixed samples, matching the scales between the modalities demands large-scale EM [1], which leads to an avalanche of data and the current quest is to identify or fingerprint biomolecules in the nm-range.

(1) *Large ROIs—data avalanche:* many initiatives try to handle and publish large imaging datasets. Our lab has pioneered placing large-scale EM maps of a variety of cells, tissues and model organisms at full resolution with open access at www.nanotomy.org: the large-scale data at the full resolution of figure 3 are available on this website. Similar initiatives are being undertaken by others and are referred to on this website. Current developments in data sharing, analysis, and interpretation—like automated recognition and machine learning software—will evolve in the next decade and solve data handling challenges. Increasingly, open access to high-content multidimensional microscopic data will pave the path for multimodal analysis of the high content datasets. Metadata definition and (online) representation of such multi-dimensional and multi-parameter datasets needs to further develop to meet international consensus.
(2) *Localization precision of many molecules and structure*: with the recent broad implementation of scanning EM in life sciences, going beyond surface characterization with secondary electron detection, but also using backscatter detectors, transmission electron detection and even fluorescence in hybrid LM/EM microscopes [1] (section 1), new opportunities to analyse tissues arise. Using the electron beam to generate signals in a scanning EM allows us to achieve lateral nanometer range localization, even if the detected signals are photons, for instance (table 1). Such as in light microscopy, axial resolution matching the lateral resolution will remain a challenge. Correlative microscopy will develop towards using more probes [19] and detectors to define the localization of molecules using endogenous signals or new probes, but not depending on fluorescence *per se*.

**Table 1.** Approaches for CLEM and ColorEM.

	In	Detected	Oppertunity	Limitation
FLM	Photons	Visible photons	Live cells, large area	Needs probes, resolution^a^
EM	Electrons	Electrons	Ultrastructure, unbiased	Limited probes, grey scale
CL	Electrons	Visible photons	HIGH resolution	Needs development
EDX	Electrons	X-rays	Endogenous and probes	Undefined molecule
EELS	Electrons	Electrons	Endogenous and probes	Undefined molecule
nanoSIMS	Ions	‘Molecules’	Endogenous andprobes	Resolution

a Except for nanoscopy; see text for details.

#### Advances in science and technology to meet challenges.

The endeavour the field made, ranging from sample preparation to multimodal microscopes with a variety of detectors, will lead to ‘correlative microscopy’ that will increasingly use the resolution that can be achieved by the electron beam, using analysis that leads to unique identifiers of molecules in EM-imaging.

#### Cathodoluminescence (CL).

In the multimodal microscopes, when electrons hit CL molecules, photons can be collected resulting in localization defined by the electron beam [20]. In addition, new analytical methods are being pioneered, which are based on using an electron beam or ion beam to achieve the required lateral localization precision.

#### Energy dispersive x-ray analysis (EDX).

Elemental fingerprinting using EDX was described in a tour-de-force way by Somlyo *et al*, 40 years ago [21], who revealed subcellular distribution of elements in muscle using spectroscopic methods. Development in EDX detectors and computer software nowadays allows (semi)routine EDX imaging of 1k  ×  1k pixel areas, to fingerprint biomolecules and probes in the context of ultrastructure (figure 3) [22].

#### Electron energy loss spectroscopy (EELS).

In parallel, elemental analysis is performed using EELS. Leapman *et al* pioneered EELS imaging in tissue to detect endogenous elements [23]. Similarly, EELS TEM allows the detection of particles enriched in certain elements targeted for labelling, such as quantum dots [24]. Recently, Tsien *et al* used lanthanides-enriched molecules that can be deposited using specific probes to perform two-color EELS, to discriminate targeted molecules and biostructures of interest [25].

#### Secondary ion mass spectroscopy at the nanoscale (nano-SIMS).

Elemental analysis using the electron beam leads to fingerprinting of which class of biomolecules are present. However, this does not typically identify molecules, like defining which protein is present. Using an ion beam instead allows the performance of nano-SIMS [26]. Thus, a map of isotope-labeled proteins can be identified at up to 50 nm resolution. The electron-beam and ion-beam techniques hold great promise to become standard tools in correlative microscopic imaging, as they allow imaging of characteristics that lead to molecular identification of biomolecules in an unbiased manner.

#### Concluding remarks.

CLEM has developed to a semi-routine technique. Major bottlenecks, like retaining fluorescence in EM-prepared samples have been overcome. Thus, a combination between nanoscopic fluorescence microscopy and EM is now feasible. CLEM will develop to make the workflow more convenient, faster and more generically available. The data sets will be larger, and protocols for reuse of data will be developed as exemplified by several initiatives to already share large-scale EM data.

The major breakthrough in correlative microscopy in the decade to come is predicted to be the generic use of multimodal microscopic imaging. Microscopists will make better use of the electron beam or ion beam, to generate signals that fingerprint or identify biomolecules and structures, either directly or indirectly, using to-be-developed probes and bypassing the diffraction limit of light microscopy. These developments will lead to multidimensional EM with a pleiotropy of signals and molecules detected at nm-scale precision and reveal many current secrets underlying the regulation of life.

### Acknowledgments

I thank my team members and Jacob Hoogenboom, Delft University of Technology, for discussions. Our work relevant to this paper is supported by the Netherlands organization for scientific research (ZonMW 91111.006; STW Microscopy Valley 12718; TTW15315).

## Super-resolution CLEM: from room temperature to cryo-imaging

### Rainer Kaufmann^1,2,3^ and Kay Grünewald^1,3,4^

^1^ Division of Structural Biology, Wellcome Trust Centre for Human Genetics, University of Oxford, Oxford, United Kingdom

^2^ Department of Biochemistry, University of Oxford, Oxford, United Kingdom

^3^ Centre of Structural Systems Biology Hamburg and University of Hamburg, Hamburg, Germany

^4^ Heinrich-Pette-Institute, Leibniz Institute of Virology, Hamburg, Germany

#### Status.

**Figure 4.**
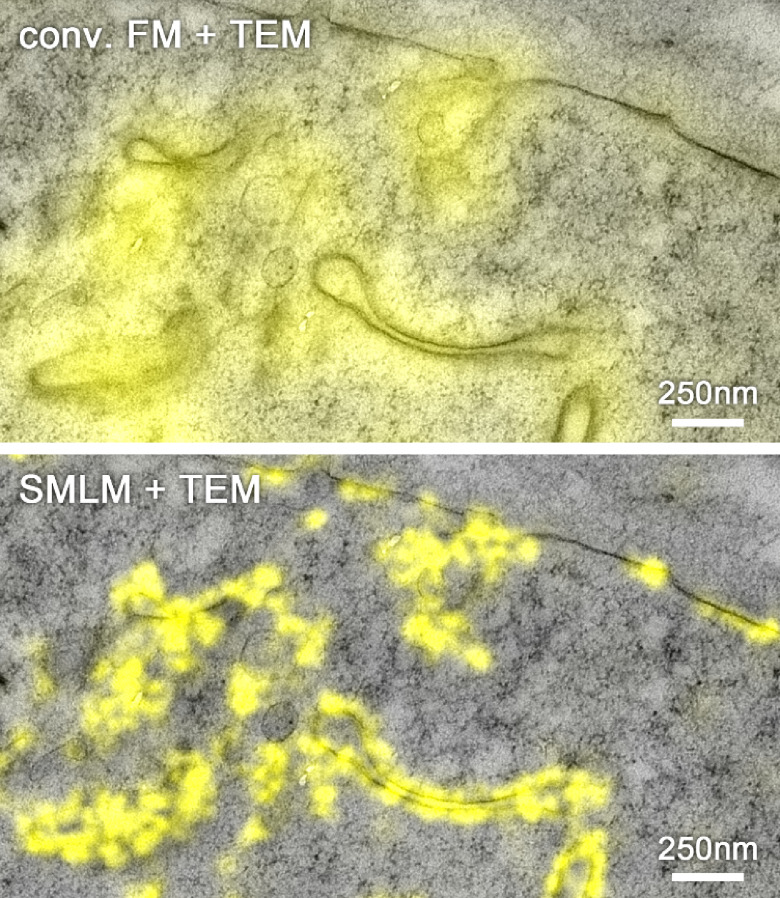
Overlay of fluorescence signal of mVenus-labelled EphA2 protein imaged by conventional FM (top) and super-resolution single molecule localization microscopy (SMLM) (bottom) with a TEM image in a freeze substituted and resin embedded HEK293T cell using a dedicated super-resolution CLEM protocol [34]. The structural resolution of in-resin SMLM was approx. 50 nm with an average single molecule localization accuracy of 17 nm. Reproduced from [29]. CC BY 4.0.

Super-resolution correlative light and electron microscopy (super-resolution CLEM) is a quickly evolving addition to the CLEM field that presents a true game-changer (see figure 4). Before, the large resolution gap between conventional fluorescence microscopy (FM) and EM did typically only allow for FM-based rough localization of areas or events of interest to be subsequently targeted by EM imaging. Despite the complementarity of both microscopy techniques, true correlative imaging was not possible before the introduction of super-resolution FM methods.

With the growing number of super-resolution methods and the plethora of EM protocols, the complexity and choices of combining both imaging modalities has tremendously increased over the past few years, since the first demonstration of super-resolution CLEM by Betzig *et al* in 2006 [27]. Microscopy hardware has significantly advanced for both imaging modalities, and the challenges in super-resolution CLEM, as Shtengel and Hess have recently pointed out [28], are mainly in sample preparation. Often, the typical protocols for EM are incompatible with super-resolution FM requirements and vice versa. Recently, various workflows addressing these limitations have been developed, enabling there now to be more possibilities for combinations of super-resolution CLEM. For example, the development of an OsO_4_ resistant photoactivatable fluorescent protein (FP) allowed the introduction of super-resolution FM compatible markers into the typical EM fixation workflow, which, in its standard protocol, destroys the fluorescence [7]. In an alternative approach, the freeze substitution and resin embedding procedure was adapted to maintain the fluorophore’s photo-switching capabilities, enabling in-resin super-resolution CLEM using standard FPs [29].

For cryo-conditions, the challenges, and hence the current status of super-resolution CLEM, are very different. While a resolution down to the Ångstrom range for biological samples is possible on the cryo-EM side, the resolution of cryo-FM is severely limited by respective technical requirements [30]. Most importantly, the lack of high-numerical aperture (NA) cryo-immersion objective lenses reduces the resolution to about half of what is achievable in conventional FM at ambient temperatures. While the feasibility of super-resolution cryo-CLEM has conceptually been demonstrated [31, 32], huge technical and photo-physical challenges [33] are currently hindering routine biological applications of this potentially very powerful technique.

#### Current and future challenges.

At ambient temperatures, super-resolution CLEM requires balancing requirements of resolution in FM, contrast in EM and structural preservation [7, 28, 29, 33, 35]. For example, Johnson *et al* [29] reported that tannic acid, the key component for preserving the photo-switching of FPs in their protocol, had an antagonistic effect on the achievable FM resolution and structural preservation of the sample. Further, due to structural changes during the EM fixation, dehydration, embedding and even imaging processing— which are typically performed after FM data acquisition—the correlation of FM and EM images can suffer a fairly large uncertainty regarding the relative positions of fluorescent labels and EM structural features [28]. Other approaches, such as the above-mentioned OsO_4_-resistant photo-activatable FP [7] or preservation of photo-switching of FPs during freeze substitution and resin embedding [29], minimize the structural changes between super-resolution FM and EM data acquisition, by allowing for imaging on specimens after EM preparation. This results in improved correlation accuracy, but imposes limits regarding the choice of suitable fluorescent markers.

**Figure 5.**
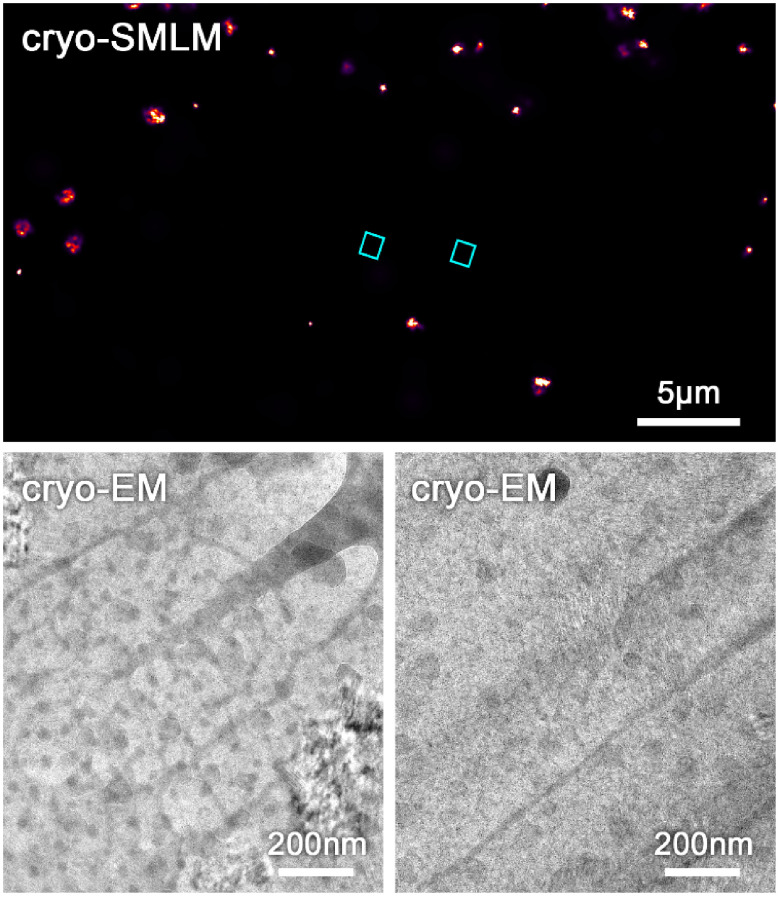
Top: cryo-SMLM of a mitochondrial protein labelled with clover in a whole vitrified COS7 cell before thinning by focused ion beam milling (for method see [12]). For cryo-SMLM a laser intensity of approx. 1.6 kW cm^−2^ was used. Bottom: cryo-EM images after thinning of the areas (blue rectangles) indicated in the cryo-SMLM image. Segregation artefacts and Bragg reflections indicate devitrification (ice crystal formation) caused by cryo-SMLM imaging.

Super-resolution cryo-CLEM, on the other hand, is currently still much more limited by technical challenges [30]. Cryo-FM suffers from the fact that, so far, no high-NA immersion objective lens has been developed for cryo-conditions. This restricts not only the optical resolution, but also the detection efficiency, that is critical for super-resolution methods. Another aspect crucial for single molecule localization microscopy (SMLM) based super-resolution FM is the ability of photo-switching of fluorescent molecules, but the underlying photo-physics is only poorly understood for cryo-conditions. So far, only SMLM methods have been used for super-resolution cryo-CLEM [31, 32]. These require a certain level of laser intensity for switching the fluorescent molecules to achieve super-resolution. Accordingly, the biggest challenge currently preventing the wider biological application is sample devitrification by local warming, resulting in the transition of amorphous ice to a crystalline form, thereby destroying the biological structures [30–32] (figure 5). Hence, so far, successful super-resolution cryo-CLEM has only been achieved by using cryo-protectants and/or formvar coated grids [31, 32], which are both not ideal for cryo-EM [30].

#### Advances in science and technology to meet challenges.

For the advancement of super-resolution CLEM under ambient conditions, improvement of the sample preparation protocols remains the most important aspect. The development of more fluorescent markers or protocols to preserve their capabilities for super-resolution imaging will make super-resolution CLEM suitable for a wider range of biological questions. Optimization of these fluorophores and dedicated protocols will, moreover, help to increase the resolution of FM. Parallel to this, it is also important to minimize structural changes, both those that might occur between FM and EM imaging, and those which might become problematic in general for the biological interpretation of the data. If the correlation accuracy becomes the limiting factor in super-resolution CLEM, there is no point in pushing the FM resolution even further, and evidently, both imaging modalities will be impaired if structural artefacts arise.

Super-resolution CLEM under cryo-conditions currently has many more areas that require improvements. The crucial parameter of SMLM super-resolution imaging is typically the photo-switching of the fluorescent markers. This is very poorly studied and understood under cryo-conditions, and is probably the factor that would have the biggest impact on the quality and usability of super-resolution cryo-CLEM. Another (often neglected) challenge is the problem of devitrification. This has also currently been the reason why stimulated-emission-depletion, requiring at least in its standard form very large laser powers, has not yet been successfully applied in cryo-CLEM applications [30]. A generally applicable solution is required, that overcomes the current limitation to subset samples, and is fully compatible with cryo-EM imaging. Super-resolution cryo-CLEM loses its justification if the advantages of cryo-EM—structural preservation and highest resolution—cannot be maintained. Encouragingly, several groups are now actively tackling these challenges and a noticeably wider community of hardware providers have become interested.

#### Concluding remarks.

Super-resolution CLEM is becoming an established method, for which current developments are focusing on making it compatible with a broader range of samples, and overcoming the limitations imposed by the choice of fluorescent markers or other constraints during sample preparation. Currently, super-resolution CLEM methods based on freeze substitution and resin embedding provide, for most biological applications, the best compromise of resolution on the FM side, and structural preservation on the EM side. In contrast, super-resolution cryo-CLEM is a technique that is just emerging and still requires significant improvements to turn it into the powerful biological tool it promises to be. If proven practically feasible for a wider specimen range, it will surely become the method of choice, due to the superior structural preservation of vitrified samples and resolution on the EM side, and might even take the full advantage of improved fluorophore properties under cryo-conditions.

### Acknowledgments

We acknowledge support from the Wellcome Trust (107806/Z/15/Z to KG and 107457/Z/15/Z to Micron Oxford), HFSP (RGP 0055/2015 to KG) and CRUK (A17721 to E Yvonne Jones).

## CLEM probes

### Paul Verkade^1^ and Lucy Collinson^2^

^1^ School of Biochemistry, University of Bristol, Bristol, United Kingdom

^2^ Francis Crick Institute, London, United Kingdom

#### Status.

In almost all imaging techniques, the selection of the right probe(s) is an essential part of the workflow. Probes are used to mark the region of interest and to reveal functional information about the biological processes under investigation. For correlative imaging, where different imaging modalities are combined in one experiment, it is possible to use a probe that is visible in one modality (e.g. fluorescence microscopy) and to track the physical location of the probe, and analyse the properties of the sample in another imaging modality (e.g. electron microscopy). However, if the probe is not visible in the second imaging modality, there will be a degree of uncertainty in the correlation. A true correlative probe must be visible in each imaging modality used. This is a challenge because light, electron, x-ray and force microscopes have fundamentally different contrast mechanisms and sample interactions.

The ideal probe will reach its target on, or in, cells and tissues with no cytoxicity and no adverse effects on ultrastructure or on the biological process under study. It will have maximal contrast in all imaging modalities, and will be small enough to localise the macromolecule to a cellular structure with an accuracy equal to the size of that molecule.

**Figure 6.**
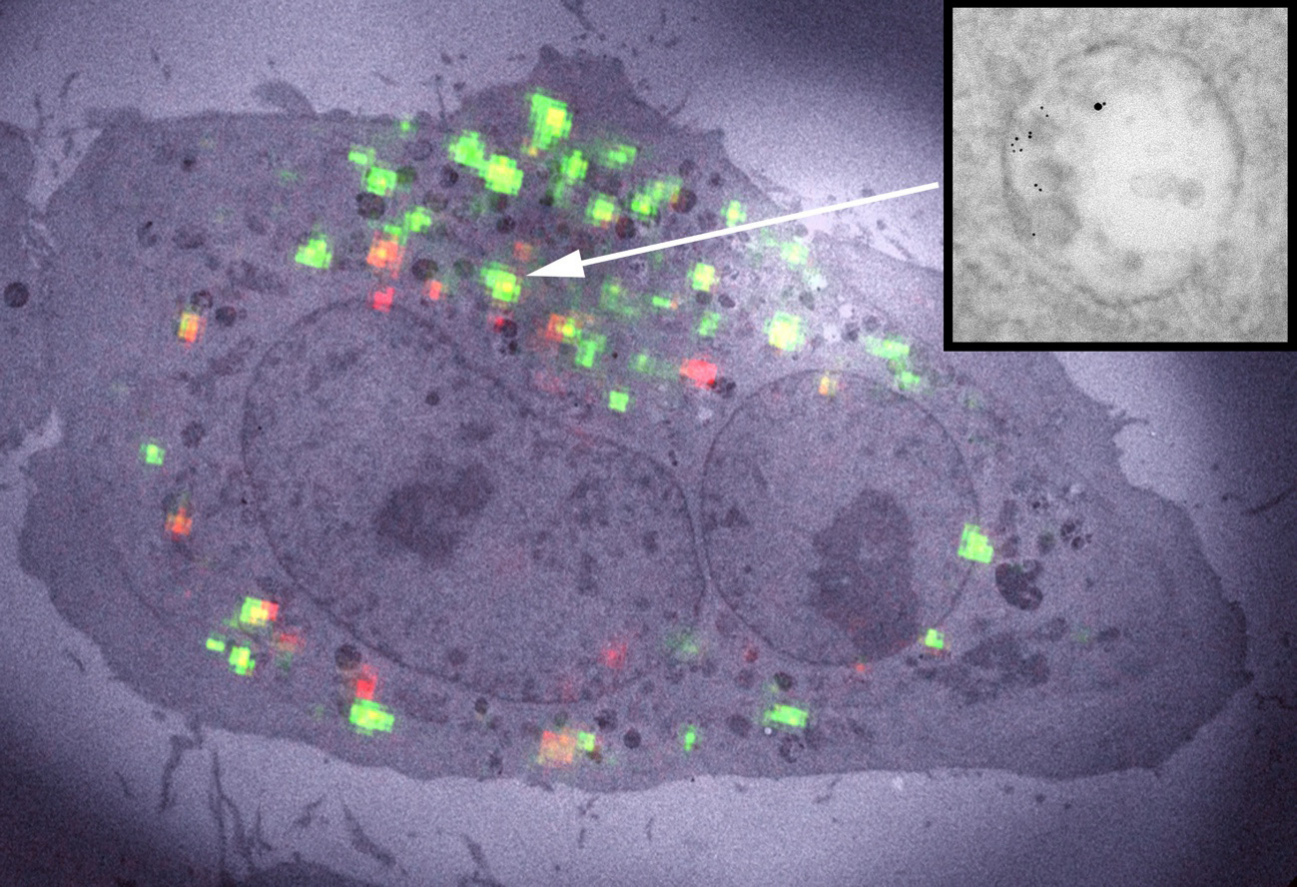
Example of a mismatch between fluorescence and gold coupling to proteins of interest. Epidermal growth factor coupled to Alexa488 and 10 nm gold particles internalised into HeLa cells shows bright fluorescence but only one gold particle at the region of interest. Transferrin coupled to Alexa488 and 5 nm gold particles, however, shows a lower fluorescence signal but numerous gold particles. Quenching of the fluorophore and coupling efficiency may play a role in this mismatch. See also [14].

Though there is a wealth of knowledge on probes and there is continuous development of new probes, the ideal universal probe is yet to be designed (figure 6) [35]. Realistically, it is unlikely that a single probe will be suitable for every different correlative imaging workflow, and researchers will need to evaluate which probe is best suited for each scientific question. There are, however, great opportunities in the field for developments, both in the probes themselves and new technologies, to detect existing probes across the different imaging modalities.

#### Current and future challenges.

To date, most correlative light and electron microscopy (CLEM) probes can be categorised into three classes:

(1) Single modality genetic probes that are compatible with sample preparation for two or more imaging modalities:
  (a) Osmium-resistant probes for fluorescence and electron microscopy—the fluorescent Eos derivative mEos4 has recently been described to withstand osmium treatment during EM processing [7]. The mEos4 molecule is therefore still fluorescent in a well-stained resin-embedded sample. In addition, it is compatible with super resolution photoactivated localization microscopy.
  (b) Fluorescent proteins—green fluorescent protein (GFP) family [5]. These fluorophores survive a mild processing regime that retains water and avoids osmium and epoxy resins during embedding. By very precise alignment of the images obtained via LM and EM using fluorescent beads, the location of GFP tagged proteins can be determined within ~100 nanometers.
(2) Single and dual modality exogenous probes:
  (a) Single fluorescent or gold probes that are photoconverted or can be visualised with multiple microscope techniques. These include the recent development of labelling non-protein molecules via click chemistry [36, 37].
  (b) Probes with a metal core and inherent fluorescent properties—lanthanides/quantum dots [38]. These probes are very well suited for CLEM experiments as they are inherently fluorescent and electron dense. But due to their relatively large size, penetration and functionalisation can be significant challenges.
  (c) Probes with a metal core conjugated to a fluorescent moiety—fluoronanogold. Fluoronanogold with its 1.4–1.8 nm gold particle conjugated next to a fluorescent molecule is much smaller than a quantum dot and therefore penetrates better but is not directly visible and requires silver or gold-enhancement.
3. Dual modality genetic probes:
  (a) Probes that can be converted from a fluorescent to an electron dense signal using photo- or chemical conversion—mini singlet oxygen generator (miniSOG). Probably superseded by APEX, mini-SOG can still be used as a CLEM probe as it is fluorescent (although not very bright) and can generate a DAB precipitate because of its singlet oxygen generating properties.
  (b) Probes with two genetic tags added together, one fluorescent and one convertible to an electron dense product—horse radish peroxidase (HRP) or APEX with a fluorescent protein (e.g. GFP) [39]. APEX is a much better SOG than mini-SOG and hence a combination with a better fluorescent molecule than mini-SOG provides a very useful combination as CLEM probes.
  (c) Probes with two genetic tags added together, one fluorescent and one metal-binding protein—e.g. metallothionein or ferritin with a fluorescent protein (e.g. GFP). To potentially improve the localisation precision of the EM marker from a precipitate to a particulate marker, APEX can be replaced by a metal-binding protein such as ferritin.

Genetic probes have an advantage because no permeabilisation of the cell membrane is required to gain access to internal structures, and so membrane integrity is preserved, which is critical for structural studies by electron and x-ray microscopy. However, expression levels must be carefully controlled, as over-expression of fluorescently-tagged molecules often disrupts the structures and processes under study. Smaller genetic probes are preferred since they are less likely to cause misfolding or aggregation effects upon expression, so a single mEos4/GFP/miniSOG construct may be preferred over a dual GFP-APEX of GFP-HRP construct.

Immuno probes avoid issues of probe expression, but varying levels of membrane disruption, using detergents (triton) or toxins (saponin, digitonin), are required to allow entry of the probes to the cell. The exception is in studies of endocytosis or phagocytosis, where probes can be fed to cells in targeted (via membrane receptors) or untargeted (via fluid-phase uptake) feeding experiments. Care must also be taken here that binding to the receptor or fluid-phase uptake does not adversely affect the trafficking pathways being studied. Access of these probes becomes increasingly difficult as the sample size increases into the tissue and organism domain. Other issues that must be considered include cytotoxicity, size and steric hindrance effects in dual labelling experiments.

#### Advances in science and technology to meet challenges.

In correlative imaging, similar to most (biological) research fields nowadays, it requires the combined effort of scientists with multi-disciplinary backgrounds to best tackle the scientific problem. Only by combining our expertise in biology, chemistry, physics and microscopy will we be able to generate better correlative imaging probes—a process that is analogous to correlative imaging in leveraging the strength of each individual microscope, and combining them in a single experiment to generate more than just the sum of each technique. Below, we highlight some of the specific areas that can contribute to such development:

(1) Advances in chemistry—targeted evolution of existing genetic probes and design of new probes to resist the sample preparation steps required to move between imaging modalities [7]; further investigation of and improvements in the production of homogenous nanoparticles of lanthanides; focused development of quantum dots as imaging probes to improve electron density whilst maintaining fluorescent intensity; development of small molecules that can enter the cell without permeabilisation or cytotoxicity and that are visible in multiple imaging modalities; investigation of new probe types with multiple contrast mechanisms (nanodiamonds, others).
(2) Advances in Molecular Biology—the use of knock-down and re-expression, especially exploiting the Crispr/CAS9 system for the expression of genetically encoded probes tagged to proteins of interest will be a major advance in controlling off-target effects of those probes.
(3) Advances in sample preparation—milder conditions compatible with preservation of probe contrast between imaging modalities [14].
(4) Advances in microscope technology—new excitation and detection regimes compatible with existing and new probes that enable ‘ideal probe’ conditions. Examples of those are the direct detection of fluorescent probes in the electron microscope using electron energy loss spectroscopy or nanoscale secondary ion mass spectrometry, Raman for probe-free element detection, or detection of metal particles using photon detection techniques such as cathodoluminescence, four wave mixing and interferometric cross-polarization microscopy [40].

#### Concluding remarks.

Most correlative imaging experiments currently link two imaging modalities. There is great potential in developing probes to link three or more imaging modalities [41], and in doing so, reach from clinical imaging (MRI, CT, PET/SPECT, fluorescence image-guided surgery) through to the molecular scale. The work must be focused around a suite of important test-case biomedical research questions to ensure that the probes are fit for purpose.

### Acknowledgments

This work was supported by the Francis Crick Institute which receives its core funding from Cancer Research UK (FC001999), the UK Medical Research Council (FC001999), and the Wellcome Trust (FC001999).

## CLEM in chemistry and catalysis

### Elke Debroye^1^, Haifeng Yuan^1^, Johan Hofkens^1^, Maarten B J Roeffaers^2^ and Kris P F Janssen^1^

^1^ KU Leuven, Molecular Imaging and Photonics, Department of Chemistry, Celestijnenlaan 200F, B-3001 Heverlee, Belgium

^2^ KU Leuven, Centre for Surface Chemistry and Catalysis, Department of Bioscience Engineering, Celestijnenlaan 200F, B-3001 Heverlee, Belgium

#### Status.

The nanoscale dynamics and complexity of heterogeneous molecular systems carry over to larger length scales, where phenomena such as diffusion, Brownian motion, crystal growth or charge carrier transport are all manifestations of stochastic processes at the molecular level. While nanoscale phenomena have a marked impact on the performance of catalytic systems [42], or the efficiency of novel energy harvesting materials [43], they are typically obscured by ensemble averaging and/or the limited time resolution of bulk characterization methods. More advanced techniques to explore the intrinsic nature of these materials at the nanoscale are therefore needed. As an example, single-molecule and/or super-resolution fluorescence (SRF) imaging [42] allow chemical activity distributions to be studied at the single particle level, in turn providing important information to understand the macroscopic performance of industrially applied heterogeneous catalysts [42, 44].

**Figure 7.**
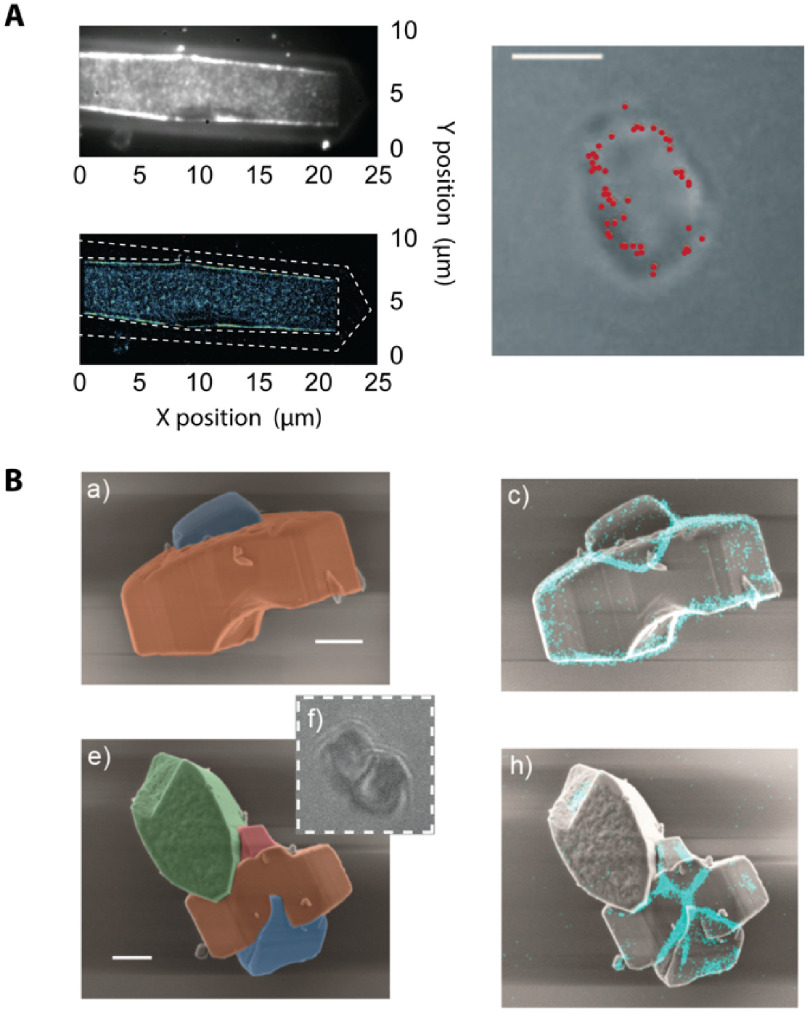
(A) When studying catalyst materials, optical microscopy can be used to visualize chemical events, e.g. using fluorogenic reagents (top left). By carefully tuning reaction conditions, the locations of chemical events can even be mapped on a particle with nanometer accuracy (bottom and right left). Unfortunately, the diffraction limit allows only a very limited amount to be derived from the particle itself (right), making it hard to correlate chemical reactivity with nanoscale structural features. Reproduced from [42] with permission of The Royal Society of Chemistry. (B) The combination of SRF imaging and EM allows individual chemical events to be correlated with ultrastructure at the single particle level. Reprinted with permission from [48]. Copyright 2017 American Chemical Society.

Although fluorescence microscopy affords spatial mapping of chemical activity or the tracking of single molecules, often with nanometre precision and high time resolution, it is less suited to provide the structural context that sets the stage for molecular events and processes of interest (figure 7). It is for these reasons that correlative light and electron microscopy (CLEM), in its various embodiments, has been successfully applied in biology to reveal structure-activity relationships [1] and is now increasingly being applied in chemistry and catalysis research.

Using CLEM, reductive N-deoxygenation of resazurin by NH_2_OH and oxidative N-deacetylation of amplex red by H_2_O_2_, both yielding the highly fluorescent resorufin, were used as probe reactions to map catalytic activity on gold nanoparticles coated with mesoporous silica (Au@mSiO_2_) [45]. The same probe reaction was used to study the photocatalytic activity at distinct surface facets of titanium dioxide (TiO_2_) nano-rods [46]. Karreman *et al* used an integrated laser scanning fluorescence microscopy and transmission EM instrument, in combination with a fluorogenic probe reaction, to map the location of Brønsted acid domains within the heterogeneous structural context of fluid catalytic cracking particles. Their integrated approach allowed changes in chemical and functional properties of the heterogeneous catalysts to be tracked over time, revealing how regions with changing levels of catalytic activity could be assigned to structural defects generated by aging induced degradation of the zeolite material [47].

#### Current and future challenges.

CLEM studies on commercially applied zeolite catalysts revealed pronounced particle-to-particle heterogeneities, and underscore the importance of defect-rich intercrystal intergrowths or post-synthesis modifications that modulate intra-crystalline diffusion or active site accessibility as major determinants of overall performance [48, 49]. However, catalyst particles are 3D entities. Whereas confocal microscopy and certain wide-field SRF modalities offer inherent 3D imaging capabilities, scanning EM (SEM) imaging typically yields a 2D view of the outer surface of the sample, preventing visualization of internal defects and structural features [50]. While focused ion beam milling can reveal the internal structure of individual catalyst particles [50], this approach does not scale well towards EM imaging of larger particle volumes.

Currently, studies on catalytic systems typically follow a consecutive approach where *in situ* fluorescence imaging of a sample is followed by *ex situ* EM imaging. While integrated CLEM systems exist, and are commercially available, further adaptations might be needed to allow truly *in situ* observation of relevant systems under ambient conditions. Reliable image registration can be achieved using either fiducials or the intrinsic photoluminescence or cathode-luminescence to determine the coordinate transformation between the SEM and fluorescence data^34^34https://github.com/KrisJanssen/QuickCLEM..

CLEM can also be applied to novel and often chemically diverse groups of materials such as, among many others, organometal halide perovskites. Featuring striking electrical and optical properties, perovskites are prime candidates for the development of next generation solar cells and optoelectronic devices [43, 51, 52]. The morphology of individual domains within condensed perovskite phases is one of the key factors determining the generation, transport, and trapping of charges [53], particularly at domain boundaries. Moreover, phase stability and domain formation in these materials is subject to various environmental factors such as oxygen and moisture, temperature, pressure and light irradiation, rendering these materials highly dynamic. To understand how perovskite composition and grain morphology influence the fate of the photogenerated charge carriers and to ultimately predict the performance of these materials in downstream applications, physicochemical and *in situ* structural analysis with high spatio-temporal resolution at the single particle level is required. This requires the expansion of existing CLEM capabilities towards highly multimodal analysis e.g.via energy dispersive x-ray spectroscopy elemental mapping or Raman scattering based imaging of chemical signatures.

#### Advances in science and technology to meet challenges.

**Figure 8.**
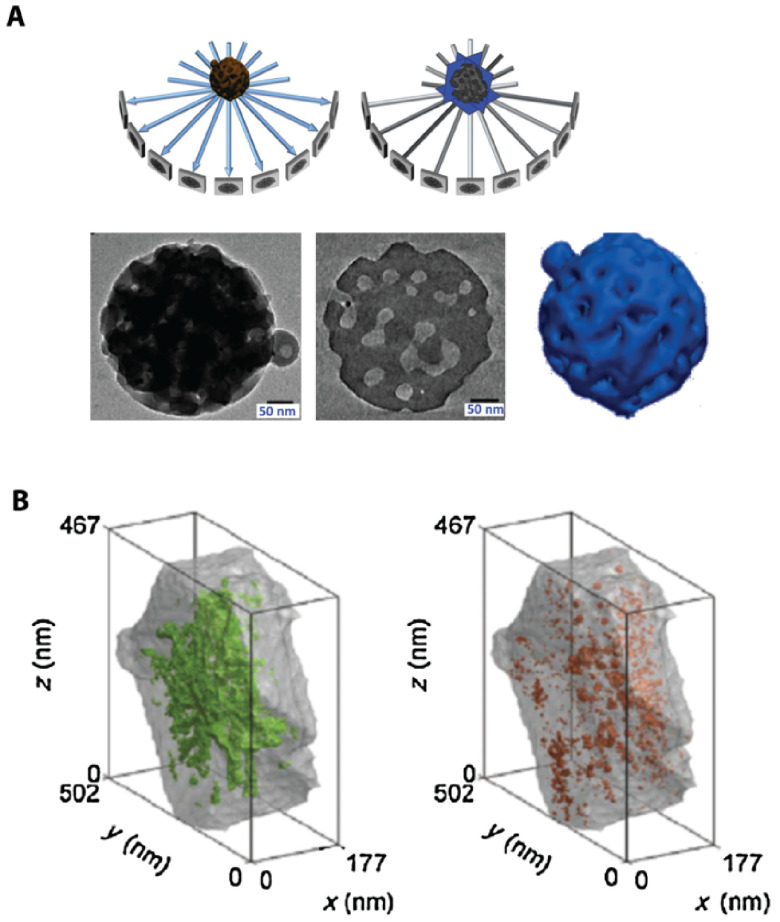
(A) The principle of ET. (B) ET allows the open (green) and closed (orange) mesopore volume in a zeolite Y crystal to be quantified. Reprinted from [54], Copyright 2015, with permission from Elsevier.

Recent improvements in EM instrumentation and computational methods allow matching 3D imaging capabilities of fluorescence microscopy. In electron tomography (ET), a series of projection images are collected at different angles of incidence of the e-beam using a high tilt sample holder (figure 8) [47]. A high-resolution 3D representation of the sample is obtained computationally (figure 8(a)) [54]. ET can be applied analytically, revealing information on the distribution of heavy elements as well as quantitatively, e.g. mapping the network of pore structures in nano-sized zeolites (figure 8(b)) [54].

Specialized liquid cells can be used to enable simultaneous light irradiation and EM imaging of samples under (near) ambient, *in situ* conditions. Using this approach, the photocatalytic activity of ZnO crystals at the crystal facet-level could be deduced [55]. Besides determining the link between photocatalytic performance and crystal facet expression, the influence of defects and structural imperfections was found to be non-negligible [56].

CLEM can be extended beyond the visualization of emissive species or chemical events. Multimodal approaches combining spectral information with luminescence lifetime analysis and localization of luminescence events reveal the dynamics of charge trapping sites, in response to the exposure of organometal halide perovskites to different atmospheric conditions, created in an environmental SEM [52]. Image correlation approaches for sub-diffraction limited fluorescence imaging can be leveraged to examine the temporal variations of luminescence intensity in CLEM which might further aid the understanding of charge carrier behaviour in complex materials [43].

In general, the potential effect of high energy electron beams on sample systems should not be neglected. Indeed, work performed in our group indicates that the photocatalytic performance can be affected [55], or that structural degradation of ZSM-22 crystals might occur under harsh EM imaging conditions. However, modern EM equipment with biased stages can help to minimize these effects. The ability of the electron beam to locally generate electric fields in materials can also be exploited to trigger certain phenomena. Recent results on perovskites have revealed how such directional electric fields lead to different degradation pathways compared to the light induced degradation [51].

#### Concluding remarks.

From this short perspective, it should become clear that with the accelerating pace of new developments in the field of correlative instrumentation, CLEM itself might soon become a concept that is far too limited to capture the richness and depth of information that can be obtained in addition to functional and structural imaging. Multimodal correlative analysis will prove essential to meet the demands of future applications in every sector of human endeavour, from catalysis and the production of green chemicals to energy production and storage, as well as in bio(medical) research. In each of these fields, a proper understanding of nanoscale phenomena is essential.

### Acknowledgments

We acknowledge financial support from the Research Foundation-Flanders (FWO, Grant Nos. G.0197.11, G.0962.13, G.0B39.15, ZW15_09 GOH6316N, postdoctoral fellowship to HY, ED and KPFJ), KU Leuven Research Fund (C14/15/053 and IDO/12/020), the Flemish government through long term structural funding Methusalem (CASAS2, Meth/15/04), the Hercules foundation (HER/11/14) and the EC through the Marie Curie ITN project iSwitch (GA-642196) and the ERC project LIGHT (GA-307523).

## Fluorescence and electron microscopy of membrane proteins within intact cells in liquid

### Diana B Peckys^1^ and Niels de Jonge^2,3^

^1^ Faculty of Medicine, Saarland University, 66421 Homburg, Germany

^2^ INM—Leibniz Institute for New Materials, 66123 Saarbrücken, Germany

^3^ Department of Physics, Saarland University, 66123 Saarbrücken, Germany

#### Status.

**Figure 9.**
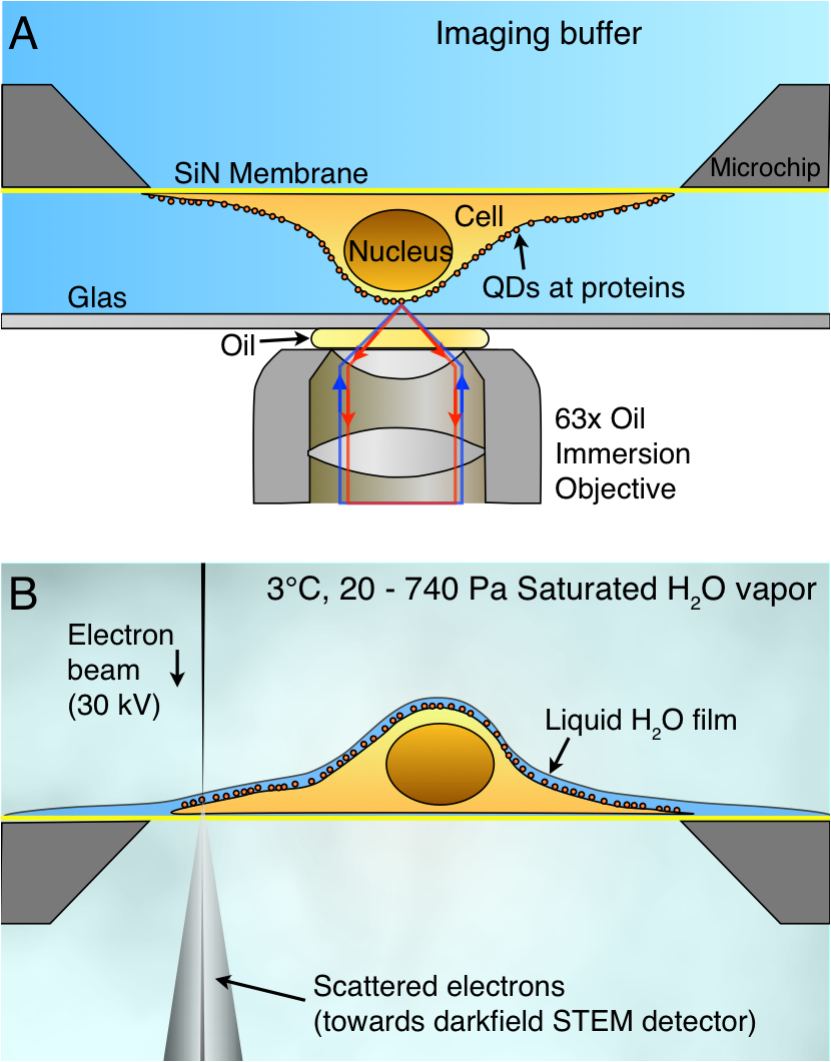
Correlative microscopy of membrane proteins in whole cells in liquid. (A) Cells are grown on a silicon nitride (SiN) membrane, supported by a silicon chip. A microchip with quantum dot (QD) labelled and fixed cells is positioned upside down in liquid in a glass-bottom dish. Fluorescence imaging is performed with an oil immersion lens on an inverted microscope. (B) For ESEM, the same microchip is positioned upright on a cooled stage and kept in a saturated water vapour atmosphere. The cells are covered with a thin layer of water. Contrast is obtained on the QDs attached to membrane proteins using the STEM detector. From [61]. Reprinted with permission from AAAS.

Electron microscopy (EM) of liquid specimens is increasingly popular in materials science, chemistry, biology, and other fields [57] to solve a wide range of so far unanswerable questions. In the life sciences, liquid-phase EM is mainly used as analytical method for studying membrane proteins in mammalian cells that are kept intact and in their native liquid environment [58, 59]. The highest resolution is obtained with scanning transmission EM (STEM). The principle relies on the atomic number (*Z*) contrast of STEM, and allows detection of specifically bound small probes, consisting of small binding peptides, or peptide tags, and nanoparticles, within several micrometers of liquid thickness and with a spatial resolution of 1–3 nm. Imaging the locations of individual subunits of macromolecular complexes is thus possible allowing, for example, to determine the stoichiometry of a protein complex. Another option is to study high-*Z* biological materials in cells such as magnetite magnetosomes [60]. The unique feature is the combination of the EM-range high spatial resolution, with the capability to study whole cells in liquid, while avoiding laborious preparation or destruction through sectioning or rupture. Liquid-phase EM can easily be combined with light microscopy (LM) to analyse protein expression levels and subcellular localization via fluorescence microscopy at the single-cell level (figure 9), thereby addressing heterogeneity in cell populations [61]. In addition, LM prior to EM makes it easy to navigate to cellular regions of interest during EM.

**Figure 10.**
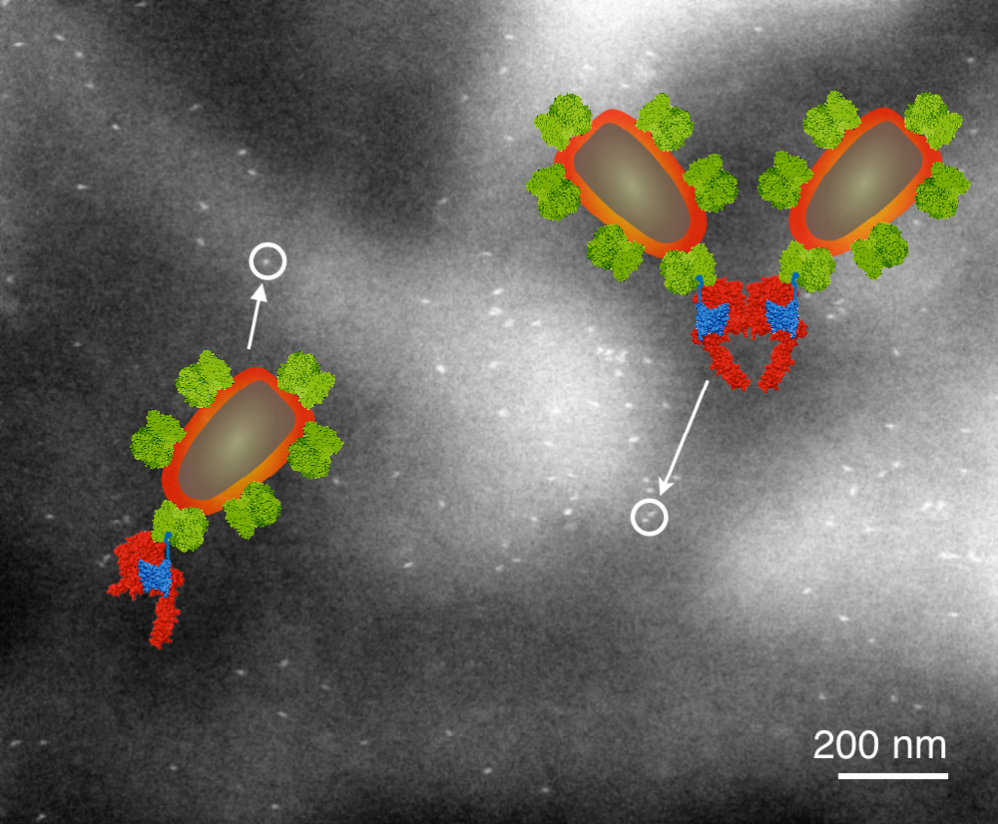
Liquid-phase ESEM-STEM image of a membrane region of a breast cancer cell showing the locations of HER2 receptors labelled with QDs. The overlay shows molecular models of the HER receptor with label consisting of an Affibody molecule (blue) coupled to a streptavidin-coated QD. The labels are attached to a monomer (left) or a homodimer (right). From [61]. Reprinted with permission from AAAS.

Liquid-phase EM adds a unique level of analytical characterization, as it gives quantitative information at a single-molecule and single-cell level, about the locations and functional state(s) of the studied proteins (figure 10). Commonly used biochemical techniques rely on extracted material from many cells, and can thus not provide information about localization. The required resolution for direct imaging of single subunits of protein complexes in intact cells is not achieved by super-resolution fluorescence techniques [62]. Other indirect optical techniques, such as Förster resonance energy transfer (FRET) and fluorescence cross correlation spectroscopy (FCCS), have their own specific limitations, such as the imposed restriction in the FRET distance (4–8 nm), which is insufficient for large protein complexes, and the need for very low expression levels in FCCS [61].

#### Current and future challenges.

Liquid-phase EM of cells comes in four different ‘flavours’, all combinable with correlative LM. Firstly, cells in liquid can be enclosed in a microfluidic chamber, sealing them against the vacuum of the electron microscope, and allowing imaging with STEM through the liquid water layer [58]. TEM is also an option, but requires the samples to be thinner than 1 *µ*m for nanometer resolution. Liquid flow can be initiated, helping to reduce radiation damage. The inherent cell thickness and bulging of the chamber in the vacuum can make it challenging to keep the liquid layer  ⩽5 *µ*m, as needed for high resolution. Typically, fixed cells are imaged, but with this method, cells alive at the onset of EM can also be studied [63]. Secondly, environmental scanning EM (ESEM) is an option for imaging cells covered under a thin liquid layer maintained in a wet environment (figure 9(b)). Using STEM detection [64] a resolution of ~3 nm is achieved [61] (figure 10). ESEM allows the fastest analysis protocol, which is particularly useful for studies involving many tens of cells. A drawback, on account of the lower electron energy of typically 30 keV versus 200 keV of regular STEM, is a lower spatial resolution and the inability to image through thicker cell regions. A third method involves the coverage of cells with an ultra-thin foil, composed of graphene sheets [65], closely fitting the cell contours, thus behaving like a flexible wrapping film [66]. It allows the imaging of the cells in liquid with 200–300 keV at the highest possible resolution. A current challenge is the cleanliness of the graphene, as it is often contaminated with small, electron-dense residues from the production. A fourth alternative, not using STEM detection, is the combination of back scatter electron detection in SEM with LM [67, 68]. The latter method does not provide as high a resolution as STEM but allows the largest flexibility in the biological experiment, because the cells are imaged in larger liquid enclosures [68] and even directly in cell culture dishes [67].

Of key importance for all liquid-phase EM methods, is their further optimization, and in particular the development of additional labels to cover a wide range of membrane protein related questions. The concept of liquid-phase EM represents a paradigm shift; it has to be clear that the aim is not to provide nanometer-scale information about the cellular ultrastructure or the protein structure as conventional EM does, although this would be possible for imaging thin cellular regions. The main value of this novel technology lies in its focus on biological and biomedical questions related to targeted membrane protein complexes, not addressable with other methods.

#### Advances in science and technology to meet challenges.

As with many pioneering techniques, it takes time for the research community to adapt liquid-phase EM into the common analytical research practices. More publications are needed demonstrating its capabilities for studying biological questions. Part of the problem is that biological research groups, with experience in and access to electron microscopes, are typically interested in high-resolution information about the structure of cells or proteins, not optimal for liquid-phase EM. On the other hand, groups with questions on membrane proteins that would be suitable for this new technique, usually are experts in methods for molecular biology and biochemistry, and may not have frequent access to EM facilities that allow experimenting with this new technique. To date, only a few groups use liquid-phase EM to study actual biological questions, for example, [61, 67]. To increase its application in life sciences, several technical issues are currently being optimized. For instance, the nanoparticle labels used so far are larger than the proteins under examination, and it is expected that a size reduction would lead to an enhanced labelling efficiency, and a higher precision in determining subunit locations. To gain information about the cellular ultrastructure in thin cellular regions, it should be feasible to use liquid STEM tomography, but this is yet to be demonstrated. Here, a limitation might be imposed by the available tilting angle, therefore low-angle tomography methods would have to be adapted. Although it would be desirable to use liquid-phase EM for live cell observations of processes at high resolution, this option is highly challenging and it requires careful control experiments. The reason is the extraordinarily large radiation intensity inherent to EM, even at the lowest possible doses of 0.1 e^−1^ Å^−2^, which is already three orders of magnitude above the dose limit for reproductive cell death. Even bacteria do not reproduce and are thus not alive according to the definition of life, even after exposure to such low doses. Yet, it is possible to study cells alive at the onset of imaging taking snapshot images of selected locations [63, 68] and it would perhaps be doable to record time lapse image series capturing biochemical processes.

#### Concluding remarks.

By offering unprecedented spatial resolution for the study of labelled subunits of macromolecular complexes in eukaryotic cells in their native liquid environment, liquid-phase EM opens an entirely new field for biological EM. The acquisition of data from many tens and even hundreds of cells in a series of experiments, comprising many tens of thousands of single-molecule data, can quantitatively address urgent problems such as cell heterogeneity in cancer. Series of whole-cell experiments can be carried out for varying experimental conditions, while still obtaining single-molecule information of endogenous proteins. Correlative fluorescence microscopy and liquid-phase EM are on their way to develop into a key microscopy methodology for future biological research.

### Acknowledgments

Ulrike Korf, David Piston, and Stefan Wiemann for discussion, and E Arzt for his support through INM. Research supported by the Deutsche Forschungsgemeinschaft SFB1027 (C7).

## Correlative light and volume electron microscopy

### Nalan Liv and Judith Klumperman

Section Cell Biology, Center for Molecular Medicine, University Medical Center Utrecht, Utrecht University, Heidelberglaan 100, 3584CX Utrecht, Netherlands

#### Status.

Correlative light and electron microscopy (CLEM) infers molecular information to an ultrastructure by uniting data from light microscopy (LM) and electron microscopy (EM). In the past decade, CLEM techniques have been used mostly to identify rare or transient cellular events by LM, and relate this to the underlying structural and cellular context by EM. Hence, CLEM has become instrumental in furthering our understanding of molecular functions in cells. So far, most CLEM applications use 2D transmission EM sections. A drawback of these approaches is that they have limited application in the *z*-axis, hampering the possibility to obtain 3D images [69]. Correlative light and volume electron microscopy (volume-CLEM) broadens the applications of CLEM to understand the function of molecules in the structural context of the 3D organization of a cell, at different organization scales: single organelles, tissues, whole cells, and eventually organs and organisms.

Recently developed volume-EM techniques that provide ultrastructural information in 3D include: serial blockface SEM [70], where the blockface is imaged repetitively after sectioning by an *in situ* ultramicrotome; focused ion beam SEM (FIB-SEM) [71], where an ion beam removes slices from the blockface; and array tomography [72], in which the serial sections from the block are placed as an array on a substrate for large area imaging in the *x*,*y* and *z* direction. In all cases, consecutive sections, or the fresh revealed surfaces of the sample block, build up a 3D stack of images, from which the volume can be reconstructed.

Volume-CLEM approaches add LM to these EM techniques, in order to facilitate the identification of a region of interest (ROI), or to provide 3D structural context information of a fluorescently localized molecule. While adding unique and essential information at the z-level, volume-CLEM also poses a significant challenge over 2D CLEM: i.e. to identifiy the same region imaged in LM back in EM in 3D. The limited correlation accuracy in z has restricted the wide applicability of volume-CLEM studies so far. However, time invested by imaging scientists to develop novel workflows is paying off, and uniting CLEM with volume-EM has now been successfully applied to visualize zebrafish development [73], brain tissue [74–77], and cellular processes like bacterial and viral infection mechanisms [78, 79].

**Figure 11.**
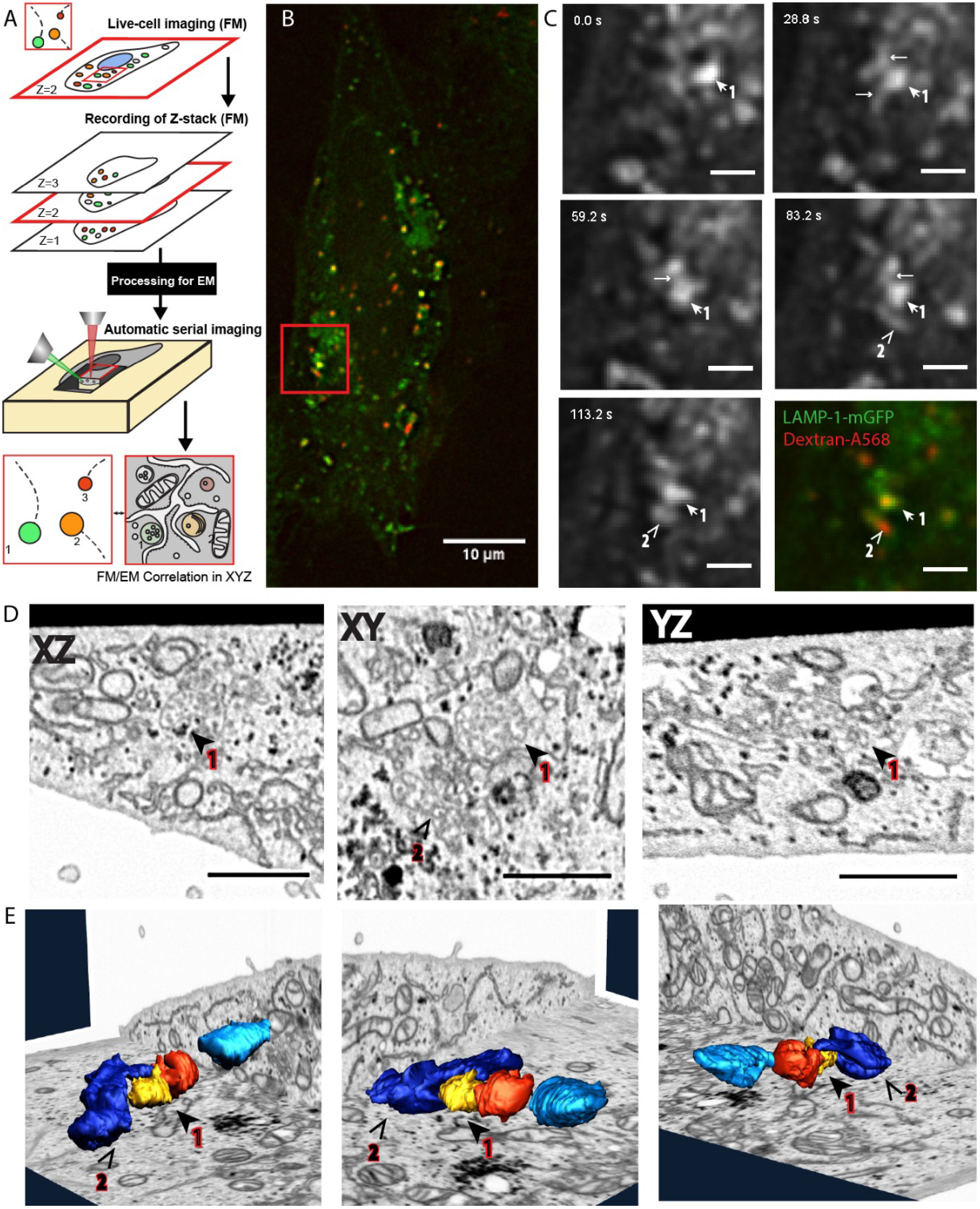
Volume-CLEM providing a direct link between live cell dynamics and 3D ultrastructure on the single organelle level [82]. (A) Schematic representation of the complete live-cell fluorescence to volume-EM workflow. (B) As an example, a fluorescence image of a LAMP-1-GFP transfected cell, incubated with Dextran-Alexa568 as an endocytic marker. (C) The cell was imaged live for several minutes, followed by *in situ* fixation. Stills show the LAMP-1-GFP spots (spot 1, 2) during 142 s of imaging. After fixation the cell is stained, embedded in resin, and imaged in FIB-SEM. (D) Shows the slices on all three viewing axes (*XZ*/*XY*/*YZ*) of the reconstructed FIB-SEM dataset containing the live-cell ROI ((B), (C) red square). Both spots 1 and 2 are classified as late endosomes based on their high number of intraluminal vesicles. (E) FIB-SEM segmentation and 3D reconstructions of spots 1 and 2; the organelles imaged in live-cell fluorescence microscopy (1,2) were segmented and correlated with reference LM data.

CLEM is most rewarding when used to relate dynamic events, only visible in live cells, to an ultrastructure (live-cell CLEM) [80, 81]. The advancements in volume-EM have enabled the evolution of live cell-CLEM to live cell-volume-CLEM, providing a direct link between live cell dynamics, functional imaging (proteins at work) and 3D ultrastructure [16, 82]. The unique combination of parameters provided by live cell-volume-CLEM can be integrated on the single cell or even single organelle level (figure 11). Live-cell-volume-CLEM thereby opens the road to literally infer kinetic information to EM images, allowing the formulation of a whole new array of questions that can be addressed with this powerful technology.

#### Current and future challenges.

Even though the tools required for volume-CLEM are now available, some challenges still prevent wide-spread routine application. Sample preparation for CLEM and volume-EM are both far from trivial and have evolved mostly independently from each other. Sample preparation approaches for volume-CLEM should therefore balance the requirements of both, and often require extensive testing of protocol variables for each sample type. In addition, a major challenge of volume-EM approaches is to perform immuno-labelling, the most widely used method for protein localization studies. Most volume-EM methods, however, are not compatible with immunolabelling throughout the sample. Pre-embedding labellings require permeabilization of cells, compromising cellular morphology. Array tomography, allowing the immunolabelling of an array of serial sections, could become the unique volume-EM method for protein localization studies in 3D, but is currently hampered by a low throughput in the sequential analysis and correlation of constitutive sections. Because of the limited immunolabelling possibility in volume-CLEM, the identification of a discrete point in 3D also becomes a significant challenge. The localization and correlation of molecules in 3D between LM and volume-EM relies mainly on fluorescent tags, and this makes the accurate correlation of 3D LM and EM data sets very crucial for volume-CLEM. A high correlation precision is currently achieved for 2D CLEM applications (sub 10 nm precision correlation), but still constitutes a challenge in the *z*-axis, because of the approximately 100-fold resolution mismatch between LM and EM datasets in the third dimension.

Structure function studies of proteins or protein complexes require quantitative imaging approaches, which is dependent on the number and volume of the sample sets that are imaged. Therefore, higher throughput and more automatized LM and EM instrumentation compatible with volume-CLEM is urgently needed. Recent studies on connectomics provide very promising examples of extended data collection [83]. Automation of instruments generates immense amounts of imaging data, and raises the problem of storage and subsequent analysis. It is becoming routine to collect raw images sizing up to hundreds of gigabytes per day, and therefore, novel strategies should be considered to manage this data flood. Moreover, processing of the collected image sets, like alignment of stacks, correlation of LM to EM and segmentation 3D rendering of structures, possesses another major challenge, and highlights the need for automation not only in instrumentation, but also in image processing and analyses. Automatized image processing and segmentation is one of the most exciting aspects of volume-CLEM, and will enable fast and user independent quantification (e.g. volume, membrane interactions) of automatically segmented structures of interest in significant sample sizes.

#### Advances in science and technology to meet challenges.

Recent innovative developments in automated volume-EM are significantly reducing the user interaction times needed to obtain large volumes of high-quality data. These include the multi beam SEM system of Zeiss which uses 61 e-beams for parallel image acquisition [84], the multi energy deconvolution imaging strategy from FEI/ Thermo Fisher, which creates isotropic imaging data by ascending the e-beam energies to target different depths [85], and the impressive 196 multi beam system with a probe size of 1 nm [86]. These instrumentation developments contribute to the generation of immense magnitudes of structural information within considerably less time, and pave the way to relating structural information to direct measures of function, as said, a major application for volume-CLEM.

Functional information can be best linked to structure when molecules are first imaged at work (live-cell imaging), and then localized in the context of their 3D ultrastructure. Considering the limited immunolabelling possibilities of volume-CLEM, the precision with which the molecules can be localized on the ultrastructure is the main challenge of volume-CLEM sample preparation. Genetically encoded peroxidase derivatives chemically convert diaminobenzidine and produce an electron dense reaction product. When fused with a fluorescent protein these constitute a promising molecular localization technique compatible with both LM and volume-EM datasets. Interestingly, recent studies showed that molecular localization studies do not necessarily depend on electron dense stains, but can be accomplished with bimodal fiducials [87]. Volume-CLEM approaches would greatly benefit from sample preparations protocols with 3D fiducials (preferably intracellular), as well as algorithms to accurately correlate LM and EM datasets in 3D. Therefore, the development of endocytic tracers which can be used as fiducials and 3D correlation software, is highly warranted. Similar probes could be used for correlation in cryo-EM approaches. Another approach for molecular localization is preserving the fluorescent signal after resin embedding. In particular, the development of fixation-resistant fluorescent probes (e.g. meos4b), in combination with LR-white or HM20 acrylic resins, is providing encouraging results. Although the sensitivity of this approach is currently modest, because of a reduction in signal due to embedding, the application is especially interesting for recently developed integrated room temperature CLEM microscopes [88, 89]. The developments of a next generation of integrated volume-CLEM microscopes (e.g. LM in a FIB-SEM), or their combination with serial section based approaches, is very favorable for fast and accurate correlative imaging for 3D analysis of cells, tissues and model organisms.

Storage and analysis of the precious volume-CLEM data is not a trivial process, and poses a challenge on the community to create common solutions. Creating public microscopy archives for volume-CLEM, e.g. electron microscopy public image archive [90], would not only provide a solution for raw data storage, but also enable scientists to analyse the deposited data and extract the information relevant for their specific scientific questions. Developments in automated image processing, e.g. semi-or fully-automatized stack alignment and automated segmentation of volume-EM datasets, are urgently needed and in the focus of attention. Full automation of the whole pipeline, i.e. collection of LM and volume-EM data, correlation, stack alignment, reconstruction and final segmentation, is the final goal and being addressed by the booming-field dedicated to EM image analysis.

Cryo-volume-CLEM approaches, in which LM and volume-EM are performed at cryogenic temperatures, avoid chemical fixation, staining, and dehydration, and allow visualization of molecular structures with Angstrom resolution. The booming cryo-EM field would greatly benefit from cryo-volume-CLEM to identify ROIs and infer molecular information to EM images. The challenges to address, in order to exploit the full potential of cryo—CLEM, are both on the cryo-LM and cryo-volume-EM sides. These include instrumental considerations about the z-resolution of cryo-LM, adaptation of super-resolution techniques, as well as understanding the spectral characteristics of fluorophores which are different at cryo conditions than at RT [91]. On the volume-EM side, the recent advances in cryo-EM of macromolecules are revolutionizing structural biology. However, there is yet no solution for EM imaging of extended volumes in cryo conditions, and the true potential of cryo-volume-EM application at the cellular level remains to be explored. Cryo-volume-CLEM approaches will greatly benefit, when both the LM and EM techniques mature and their resolutions in all 3D will match better.

#### Concluding remarks.

Biological systems encompass many organization scales, i.e. molecules, organelles, cells, tissues and organs, in healthy and disease conditions. The complexity of these systems is based on delicate balances, which depend on much more than a simple sum of the characteristics of small entities. It is increasingly appreciated that biological systems should be studied and evaluated in higher content, and by investigating the system as a whole, rather than a reductionist approach focused on simplification. Investigating the massive dynamic interplay between millions of molecules even in a single cell, requires high-content and high-resolution imaging techniques, which can relate molecular dynamics to the ultrastructure. This makes volume-CLEM approaches indispensable for paving the way to novel biological breakthroughs, in which the identification of tagged macromolecules, analysis of their functions within living cells and tissues, and their ultrastructure, are assessed in an automatized quantitative manner.

### Acknowledgments

We thank the members of our group, especially Job Fermie, for input and discussions.

## Force spectroscopy and single molecule fluorescence microscopy: unpacking single viruses

### Iwan A T Schaap^1^ and P J de Pablo^2^

^1^ SmarAct GmbH, Schütte-Lanz-Str. 9, D-26135 Oldenburg, Germany

^2^ Dpto. Física de la Materia Condensada Universidad Autónoma de Madrid 28049-Madrid and Instituo de Física de la Materia Condensada IFIMAC, Universidad Autónoma de Madrid 28049, Madrid, Spain

#### Status.

The emergence of atomic force microscopy (AFM) has made it possible to observe biological structures with sizes that are well beyond the limits of conventional optical microscopy. Because the technique works in liquids, samples can be studied under conditions that are representative for their physiological environment. Furthermore, AFM based force spectroscopy is intensively used for the mechanical manipulation of biological matter. Initial experiments, originating from physics laboratories, demonstrated that proteins shells like microtubules and viruses exhibit a clear elastic response at small deformations followed by breakage events at higher forces [92, 93]. The relevance of such mechanical characterization is evidenced by the acceptance by the biological and biomedical communities and has, for example, led to the discovery of clear correlations between the mechanical state of a virus and its infectivity [94, 95]. Further interest for mechanical measurements is generated by the evolving insight that forces play critical roles in biological processes, ranging from the unpacking of viruses at nuclear pore complexes to controlling cell differentiation [96, 97].

**Figure 12.**
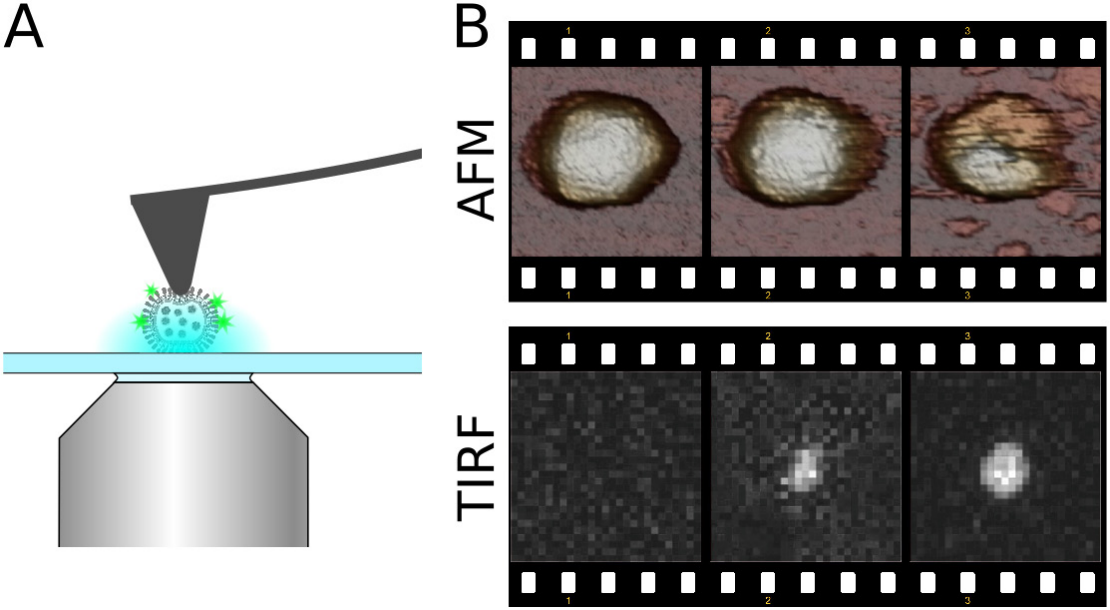
(A) A schematic of a simultaneous force spectroscopy and single molecule fluorescence experiment on a virus. An AFM tip is used for both imaging and manipulation purposes at the nanoscale. The molecular response is simultaneously tracked by imaging fluorescent markers (green) that are excited by an evanescent wave (blue). The microscope objective is shown in grey. (B) An example from a virus before and after mechanical manipulation. Top row: AFM scans (200  ×  200 nm), showing the disassembly of a virus capsid. Bottom row: fluorescence images (4  ×  4 *µ*m) showing the release of YOYO-1 labelled DNA. The DNA remains invisible in the AFM scans because it is not immobilized to the surface.

An essential condition for AFM is the immobilization of the sample to the surface, which prevents the observation of diffusive processes occurring in solution. This is unfortunate because the majority of biological processes rely on diffusion, and thus remain out of reach for AFM. To take the application of AFM to the next level, it is essential to surpass this limitation. AFM force spectroscopy has been combined with fluorescence microscopy to study protein unfolding [98] and we have recently combined single molecule fluorescence with AFM imaging and force spectroscopy, to track the release of viral DNA upon mechanical stimulation of a 100 nm small virus, thus combining the complementary benefits of each of the techniques (see figure 12) [99]. Only when the simultaneous operation of AFM based force spectroscopy and single molecule fluorescence microscopy finds its way out of the physics laboratories, will it be able to fulfil its full potential for life science research.

#### Current and future challenges.

When operated in liquid under low force conditions, AFM can resolve biomolecules with nanometer resolution. In addition, by pulling or pushing with the AFM tip, the mechanical response of the sample can be probed, which provides a method to better understand its structural organization. This combination of nanometer-resolution imaging and piconewton-resolution force spectroscopy makes AFM a seemingly ideal tool for studying biological processes at a single protein level. However, to obtain such high resolution, the sample has to be robust enough to withstand the forces exerted by the scanning tip and remain stationary while acquiring the data. Both aspects seriously limit the number of biological processes that can be studied with AFM. Additionally, AFM imaging has a rather poor discriminating power—most proteins will look like nanometer sized blobs, which makes their identification difficult in more complex samples. Although impressive progress has been made with high-speed AFM towards the observation of moving objects, it remains challenging to visualize biological processes when their dynamics are not slowed down. Imaging of freely diffusing specimens is impossible and will only lead to an increased noise level as such objects intermittently collide with the tip or cantilever. To overcome these limitations, it is a logical step to combine AFM with optical microscopy. To this end, AFMs are almost routinely placed on top of inverted optical microscopes to perform correlative studies, i.e. where AFM and optical data are obtained one after the other. Performing AFM and fluorescence simultaneously requires a drastic isolation of the AFM from mechanical vibrations that are introduced by the added microscope parts. Especially during force spectroscopy experiments, nanometer vibrations will translate in force fluctuations of tens to hundreds of piconewton. Typically, the AFM heads on inverted optical microscopes are fairly large, which leads to a large mechanical loop to the sample. This increases drift and lowers the resonance frequencies of the system. The resulting vulnerability to vibrations hampers nanometer and piconewton resolution. In addition, vibrations and thermal drift are induced by illumination sources and cameras which often contain a cooling fan. In part, these noise issues can be controlled by placing the instrument on an active isolation table within an acoustic and temperature controlled enclosure. This, however, leads to complicated and costly instruments that remain out of reach for most, except some specialized laboratories. Even so, their performance remains inferior to the stand-alone AFMs they are based on.

At the latest, when AFM and optical microscopy are combined, it becomes clear that both techniques have contradicting demands on sample preparation. AFM is performed on samples immobilized at the surface, at concentrations of tens per square micrometer, while florescence microscopy does not require immobilization and is generally performed at lower sample concentrations.

#### Advances in science and technology to meet challenges.

All of the technology needed to address the aforementioned challenges is available. Below, we list the main requirements to achieve simultaneous AFM based force spectroscopy and single molecule fluorescence, for studying bio-macromolecules in the context of life science, with sizes below the diffraction limit.

(1) *Integrated instruments with less noise*. (i) To reduce the effects of the mechanical loop between tip and sample, it is key to use a stable design (thermally and mechanically), which can be achieved by reducing the size of the AFM as much as possible [100]. (ii) In combined instruments, multiple piezo scanners and positioning stages are required to move the AFM, with respect to both the sample and the optical microscope. These need to be arranged and controlled such that the positioners that are not used for AFM will also not introduce vibrations during AFM measurements. A notorious example is the closed-loop piezo objective scanner used for focussing that can couple vibrations into the sample through the immersion oil. iii) Optical imaging does not require physical contact between the sample and the microscope and this should be exploited. In a minimal configuration, only the oil-immersion objective and a short-range focussing rack need to be implemented in the AFM stage. All other components (lenses, filters, cameras, lasers) can be mechanically uncoupled from the AFM sample stage [99].
(2) *Limit artefacts caused by the AFM tip in the optical detection volume*. During force spectroscopy measurements, the AFM tip and cantilever will be very close to the sample and thus scatter light, which can dramatically increase the fluorescence background signal [101]. Fluorescence excitation based on a total internal reflection scheme is ideal, as this limits the exited region to about 100 nm from the microscope cover slip and thus leaves the AFM cantilever and most of the tip in the dark. Confocal microscopy is, for this reason, much harder to combine with AFM.
(3) *Fluorescence super-resolution techniques*. The resolution gap between AFM and optical microscopy is more than two orders of magnitude. Confocal microscopy based super-resolution techniques are, for the reason mentioned under the previous point, less suitable, but techniques based on the accurate localization of single fluorophores [102] such as stochastic optical reconstruction microscopy (STORM) [103] can help to further increase the information that can be obtained during the measurements. A simple approach to obtain fluorescence information beyond the diffraction limit during force spectroscopy was applied during the unpacking of single viruses [99]. More advanced approaches like STORM will offer higher spatial resolution but at the cost of temporal resolution.
(4) *Sample preparation*. Studying biological processes with diffusive factors requires strategies in which only a subset of the components are immobilized to the surface, while other components remain free to diffuse [104]. A specific fluorescence response can be achieved with fluorophores that respond to mechanical or optical events. This can include Förster resonance energy transfer pairs and caged compounds, but also intercalating dyes that gain access to DNA upon mechanical stimulation [99].

#### Concluding remarks.

AFM can greatly benefit from the integration with the current state-of-the-art optical microscopy approaches. Optical detection is especially suited for identification purposes and for those parts of a sample that are soft or highly mobile. AFM imaging provides the nanometer resolution context for the localized mechanical manipulation of the sample. Once the simultaneous combination of AFM and single molecule fluorescence has reached the maturity required for its employment by biological laboratories, its application is not limited to studying relatively simple systems, like the cargo release by viruses. Indeed, an opportunity will be created to study multifold biological processes that are incompatible with current AFM approaches.

### Acknowledgments

P J de Pablo acknowledges projects FIS2014-59562-R, FIS2015-71108-REDT, Fundación BBVA and the ‘María de Maeztu’ Program for Units of Excellence in R&D (MDM-2014-0377).

## High-speed atomic force microscopy and light microscopy

### Toshio Ando

Nano Life Science Institute (WPI-NanoLSI), Kanazawa University, Kanazawa, Japan

#### Status.

Structural biology has solved the atomic structure of many proteins, but all revealed are static snapshots. Single-molecule biophysics has recorded the dynamic action of proteins but only in an indirect way of observing optical markers attached to the molecules. Therefore, it has been a desire to acquire a means to directly observe protein molecules in dynamic action. High-speed atomic force microscopy (HS-AFM) was developed to make this long-awaited observation possible. The state-of-the-art HS-AFM system allows us to image protein molecules at 10–20 frames s^−1^ (fps), without disturbing their function. Its spatial resolution is typically 2–3 nm and ~0.15 nm in the lateral and vertical directions, respectively. Despite this moderate spatiotemporal resolution, HS-AFM holds a unique and pivotal position among various tools for biological research. It is only HS-AFM that allows the simultaneous assessment of structure and dynamics of single molecules.

**Figure 13.**
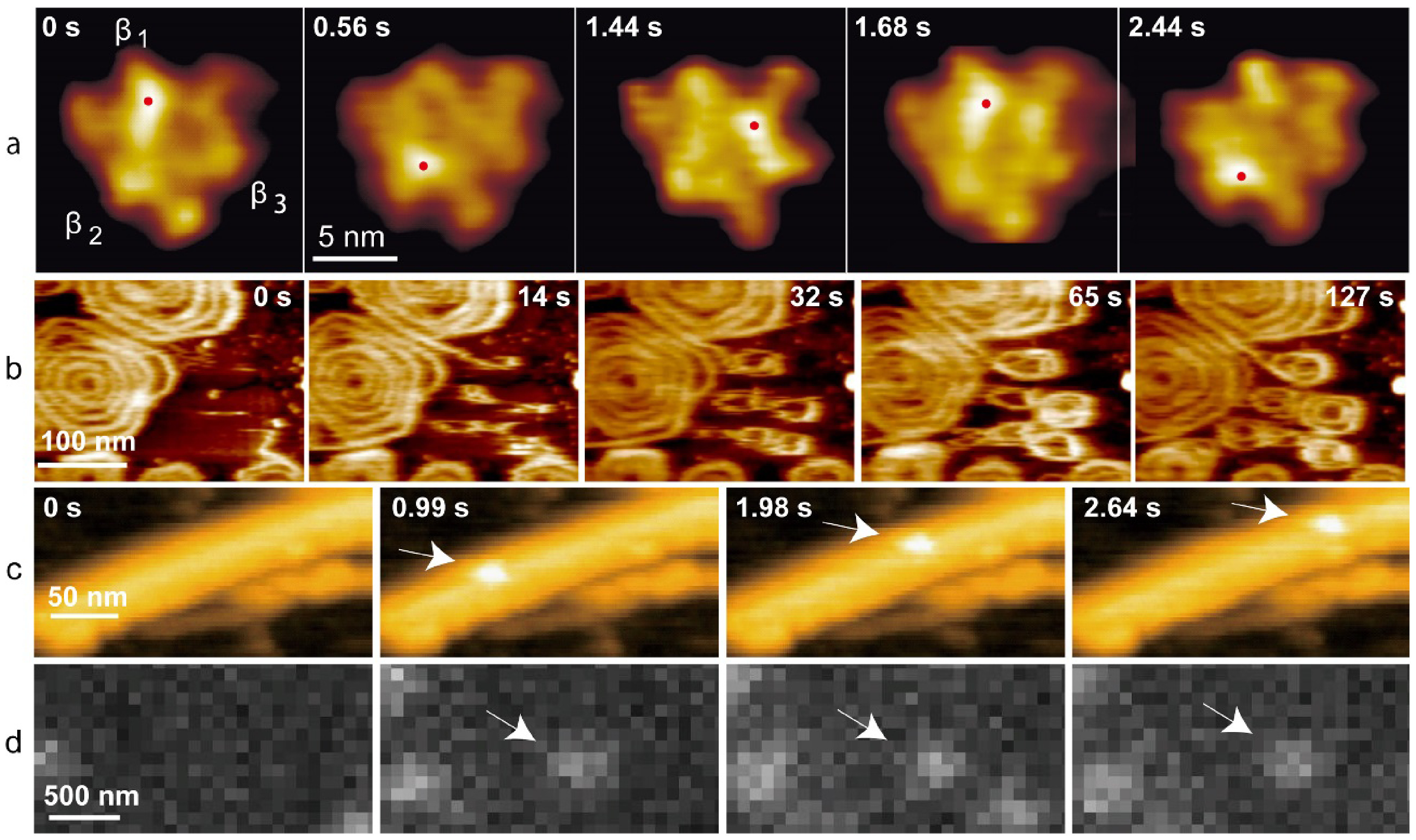
(a) HS-AFM images of *α*_3_*β*_3_ subcomplex of F_1_-ATPase undergoing conformational changes in the presence of adenosine triphosphate (ATP). The height of a nucleotide-free *β* subunit is larger than those containing ATP or adenosine diphosphate. The highest pixel positions marked with red dots shift counterclockwise. At 1.44 s, the *α* subunit adjacent to the nucleotide-free *β* subunit appears higher than the *β* subunit. Imaging rate, 12.5 fps. (b) HS-AFM images showing spiral filament formation by polymerization of the ESCRT-III protein Snf7 on a supported lipid membrane. Imaging rate, 3 fps. (c), (d) Simultaneously captured HS-AFM (c) and total internal reflection fluorescence microscopy (d) images of Cy3-labeled chitinase A (arrows) moving unidirectionally on a chitin microfibril. Imaging rate, 3 fps.

A variety of purified protein systems have been successfully visualized by HS-AFM [105]. Even protein molecules on the surfaces of live bacteria [105] and nuclei [106] have also been visualized. Moreover, the development of fast, wide-area scanners has given HS-AFM the capability to visualize live eukaryotic cells [107]. The HS-AFM movies of proteins have provided new mechanistic insights that could not have been reached in other ways, as exemplified by the discoveries of an adenosine triphosphate (ATP)-independent generation of intramolecular tension and powerstroke in myosin V [108], rotary propagation of chemical and conformational states in rotor-less F_1_-ATPase (figure 13(a)) [109], unidirectional motion of cellulases hydrolysing cellulose fibers [105], and the formation of ESCRT III spiral filaments with unnatural curvatures, leading to membrane buckling, budding and abscission (figure 13(b)) [110]. Besides, HS-AFM was demonstrated to be able to visualize the very thin and highly flexible structure of intrinsically disordered proteins [105]. As illustrated by these examples, the improvement from static/indirect to dynamic/direct observation can advance in depth and detail our understanding of how proteins function.

And yet, HS-AFM still has plenty of room for improvement and functional expansion towards various directions, one of which is hybrid HS-AFM/light microscopy (LM). LM, particularly fluorescence microscopy, provides information complementary to that acquirable with HS-AFM.

#### Current and future challenges.

This hybrid microscopy is required for the following purposes: (i) positioning the AFM tip on a region of interest within a large object (e.g. eukaryotic cells) visualized with LM, (ii) localizing specific molecules labelled with fluorophores within an overlaid topography image of complicated biological architectures, (iii) observing interaction of HS-AFM-visualized protein molecules with other molecules invisible for HS-AFM, such as small ligands and encapsulated substrate proteins, (iv) detecting minute changes in protein molecules that are only detectable with fluorescence energy transfer, and (v) observing protein molecules under an external force applied via optical tweezers.

The purposes (i) and (ii), not requiring simultaneous HS-AFM/LM observations, are already accomplished [107, 111]. A hybrid HS-AFM/fluorescence microscopy (including total internal reflection fluorescence microscopy) is already used (figures 13(c) and (d)) together with a wide-area scanner that facilitates a post-experiment positional correlation of AFM/fluorescence microscopy images [111]. Far-field super-resolution fluorescence microscopy, such as stimulated emission depletion microscopy and single-molecule localization microscopy, is already combined with conventional AFM [112]. Combining with HS-AFM will be readily accomplished.

The purposes (iii)–(v) impose challenges. In current HS-AFM, the sample stage is scanned. When this system is combined with LM, the LM image oscillates, and more seriously, the optical path of LM is blocked by the cantilever. In tip-scan HS-AFM, the laser beam focused on the cantilever has to be steered quickly to track the cantilever motion, at least in the lateral direction. A 2D mirror tilter has been recently developed, which can perform this tracking at 1 kHz, up to 20 *µ*m [111].

One of the other challenges is to correlate between HS-AFM/LM images captured in synchrony. This is because of a large difference in the field of view; the scan range in HS-AFM imaging of protein molecules is typically ~100  ×  100 nm^2^, while the field of view in far-field LM is ~30  ×  30 *µ*m^2^. Moreover, the spatial resolution gap between HS-AFM (2–3 nm) and conventional LM (200–300 nm) also makes this correlation investigation difficult. Besides these issues, the susceptibility of the cantilever to strong pulsed laser illumination in LM, or to high-power YAG-laser illumination in optical tweezers, is another issue to be considered in the hybrid system.

#### Advances in science and technology to meet challenges.

HS-AFM can visualize dynamic conformational changes of protein molecules induced by ligand binding/release. However, it is often not easy to judge whether or not visualized conformational changes are really those induced by ligand binding/release. Simultaneous HS-AFM/fluorescence microscopy observations facilitate this judgement, when fluorescent ligands are available. However, correlation identification between HS-AFM/fluorescence images is also required.

The achievable single-nanometer resolution of far-field super-resolution fluorescence microscopy is incompatible with high temporal resolution. Moreover, most of the techniques rely on strong pulsed laser illumination. These features are inadequate for materializing simultaneous HS-AFM/fluorescence microscopy imaging.

**Figure 14.**
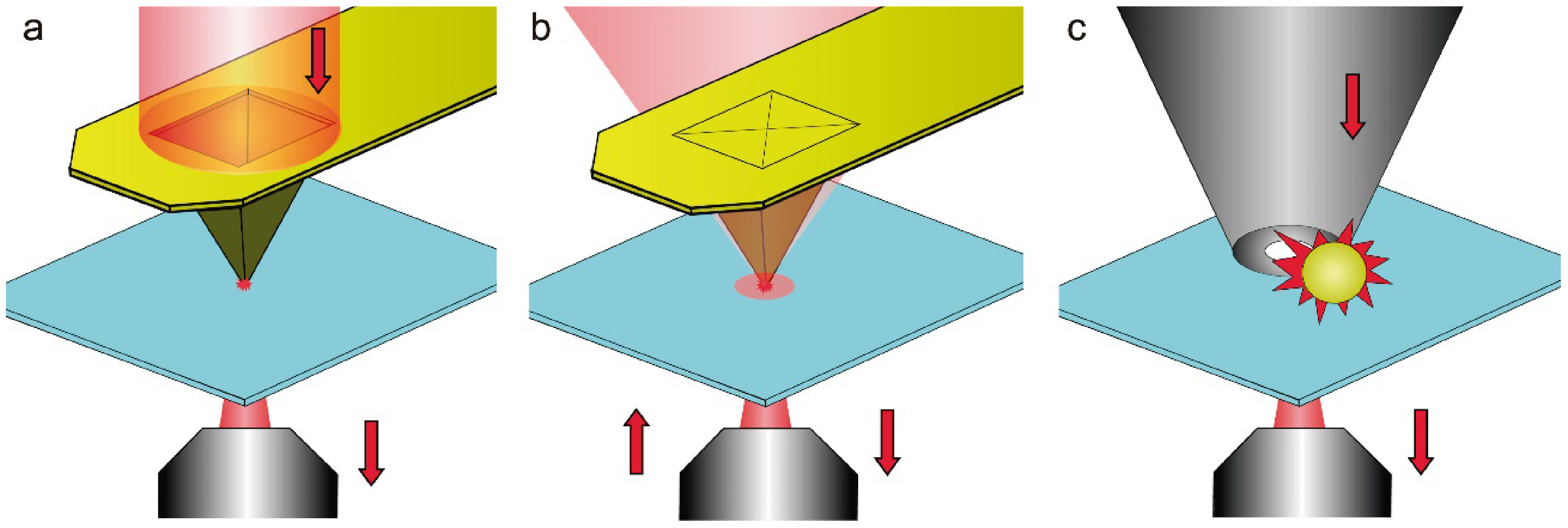
Various configurations of cantilever-based NSOM. (a) Aperture NSOM relying on a specially designed cantilever chip. The tip has a small optical opening at the apex, where a localized evanescent light is produced. (b) Aperture-less NSOM based on a metal-coated cantilever tip that acts as a local electromagnetic antenna (plasmon resonator). (c) NSOM combining a small aperture with an electromagnetic nano-antenna. The nano-antenna is a metal particle with the shape of a bow-tie, rod, sphere or dumb-bell.

Near-field scanning optical microscopy (NSOM) also beats the diffraction limit and therefore can be a candidate to be used in the HS-AFM/fluorescence microscopy hybrid system. NSOM uses either aperture probes for local illumination or aperture-less probes for local optical detection/illumination [113]. AFM/NSOM hybrid systems already exist, where the AFM cantilever (metal-coated) tip is also used as an aperture (figure 14(a)) or aperture-less (figure 14(b)) probe for NSOM. The simultaneous, pixel-by-pixel acquisition of the optical and AFM signals, at nearly identical sample positions, facilitates finding a correlation between the two overlaid images. In aperture NSOM, the spatial resolution achieved so far is 50–100 nm, while it is 10–25 nm in aperture-less NSOM. However, in aperture-less NSOM, the optical contrast is deteriorated by background signals produced by the far-field or evanescent illumination of a wide area. Recently, this problem has been overcome by combining an aperture probe with a metal (mainly gold or silver) particle, having special shapes, as an optical nano-antenna (figure 14(c)); the strong plasmon resonances localized around the particle are used as a local illuminator [114].

To combine this plasmonic aperture NSOM with HS-AFM, micro/nano-fabrication techniques have to be established, to produce short cantilevers with both an aperture probe and nano-antenna. Moreover, a fabrication technique to produce nano-antennas under precise size/shape control is also required. When an HS-AFM/NSOM hybrid system is used to observe the interaction between a protein molecule and a fluorescently labelled ligand (e.g. ATP) molecule, high spatial resolution, 5–10 nm, will often be required for NSOM, especially for homo-oligomeric proteins. This high spatial resolution is already reported but has not become routine. This is primarily because of a lack of techniques for the mass production of NSOM probes with identical properties.

HS-AFM visualization of single protein molecules under external force can provide new mechanistic insights into their dynamic processes, such as unfolding or refolding processes, and processes undergone by force-responsive/force-generating proteins (e.g. mechano-responsive receptors/channels, mechano-sensor kinases, cell adhesive proteins, and motor proteins including DNA helicases and viral DNA packaging proteins). Rather than constructing an HS-AFM/optical tweezers system, its operation is challenging because the operator has to handle HS-AFM, LM and optical tweezers, while avoiding cantilever-YAG laser beam contact. Therefore, an automation control system is important in facilitating this complicated operation.

#### Concluding remarks.

Truly useful and innovative correlation microscopy of HS-AFM/LM should allow simultaneous fast recording of the two modalities of images with well-defined pixel-by-pixel correlation. To materialize this imaging, higher resolution NSOM has to be routinely achieved. At the same time, micro/nano-fabrication technology has to be established to produce small cantilevers with a nano-illuminator for plasmonic NSOM. When the objects to be visualized by NSOM in this correlation microscopy are small ligand molecules, we will encounter an additional challenge to synthesize fluorescent functional ligands. The HS-AFM/optical tweezers hybrid system will provide a new opportunity to study the unfolding/refolding processes of proteins and directly observe protein molecules responding to an external force, exerted at a specific locus in a given direction. Although it is difficult to foresee the future progress in these hybrid systems, their materialization will surely lead to new important discoveries of proteins.

## Traction force microscopy

### Ulrich S Schwarz^1^ and Christian Franck^2^

^1^ Institute for Theoretical Physics and BioQuant, Heidelberg University, Heidelberg, Germany

^2^ Department of Mechanical Engineering, University of Wisconsin-Madison, 1513 University Ave, Madison, WI 53706, United States of America

#### Status.

**Figure 15.**
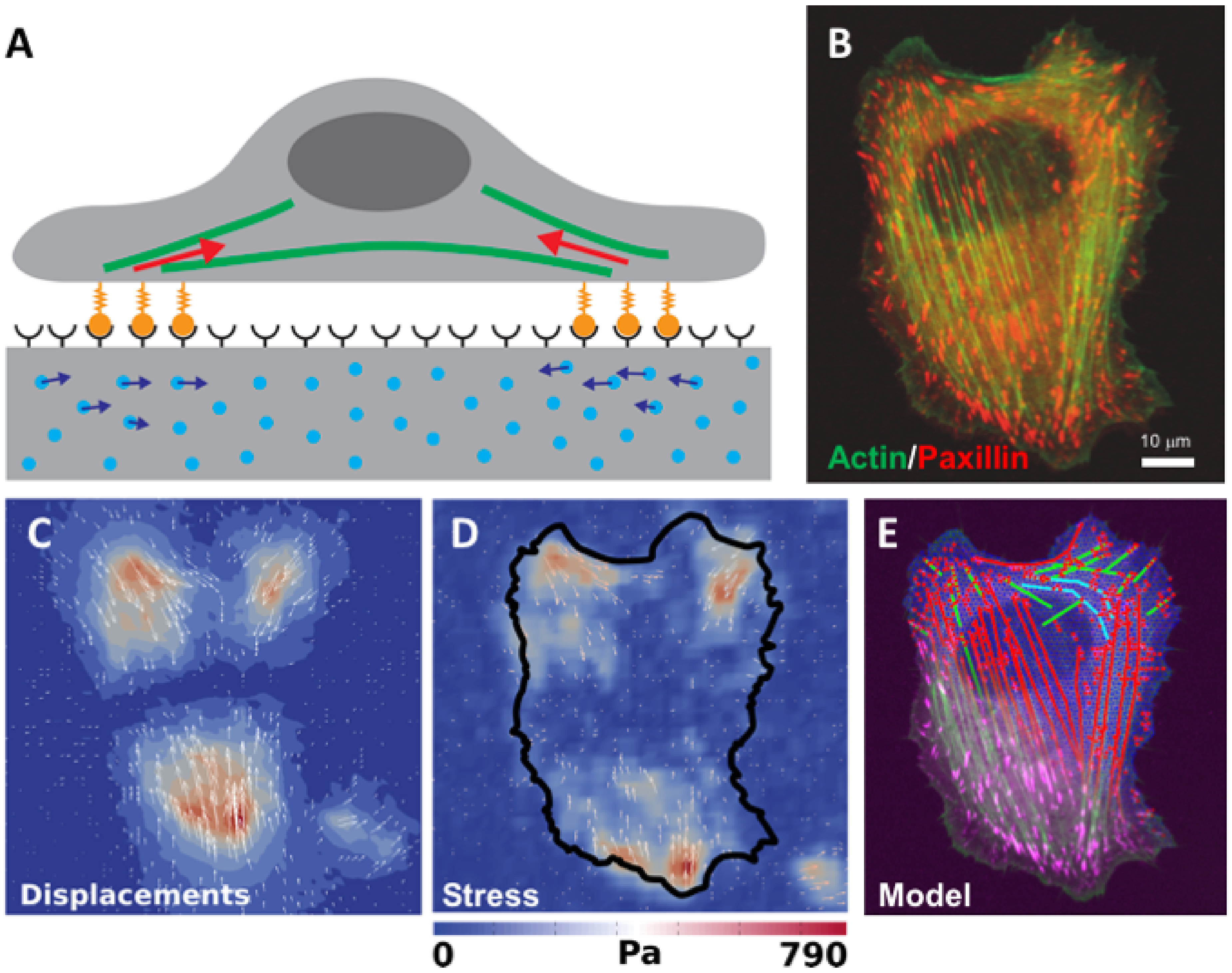
2D TFM. (A) Movement of marker beads in the flat elastic substrate are converted into an estimate of the cellular forces at adhesion sites. (B) An adherent U2OS-cell has many adhesion sites (marked by paxillin) and actin-based stress fibers. (C) Image processing is used to track substrate displacement. (D) Elasticity theory is used to calculate the traction stresses. (E) The image data can be used with a mechanical model for the cell to estimate forces in individual stress fibers. Reproduced from [121]. CC BY 4.0.

Traction force microscopy (TFM) was originally developed in the late 1990s as a technical procedure to convert the deformations of a flat elastic cell culture substrate (typically made from polyacrylamide gel) into an estimate of cellular traction forces, using the equations of elasticity theory [115]. Because cells on flat substrates spread very thin and pull on their substrate, mainly tangentially, it is often sufficient to restrict the force reconstruction to two dimensions (‘2D TFM’) (figure 15). Early efforts in the field focused on improving the reliability and efficiency of this method, for example, by performing the calculations in Fourier space, introducing regularization techniques from control theory, and by improving the image processing methods required to track substrate deformations [126].

**Figure 16.**
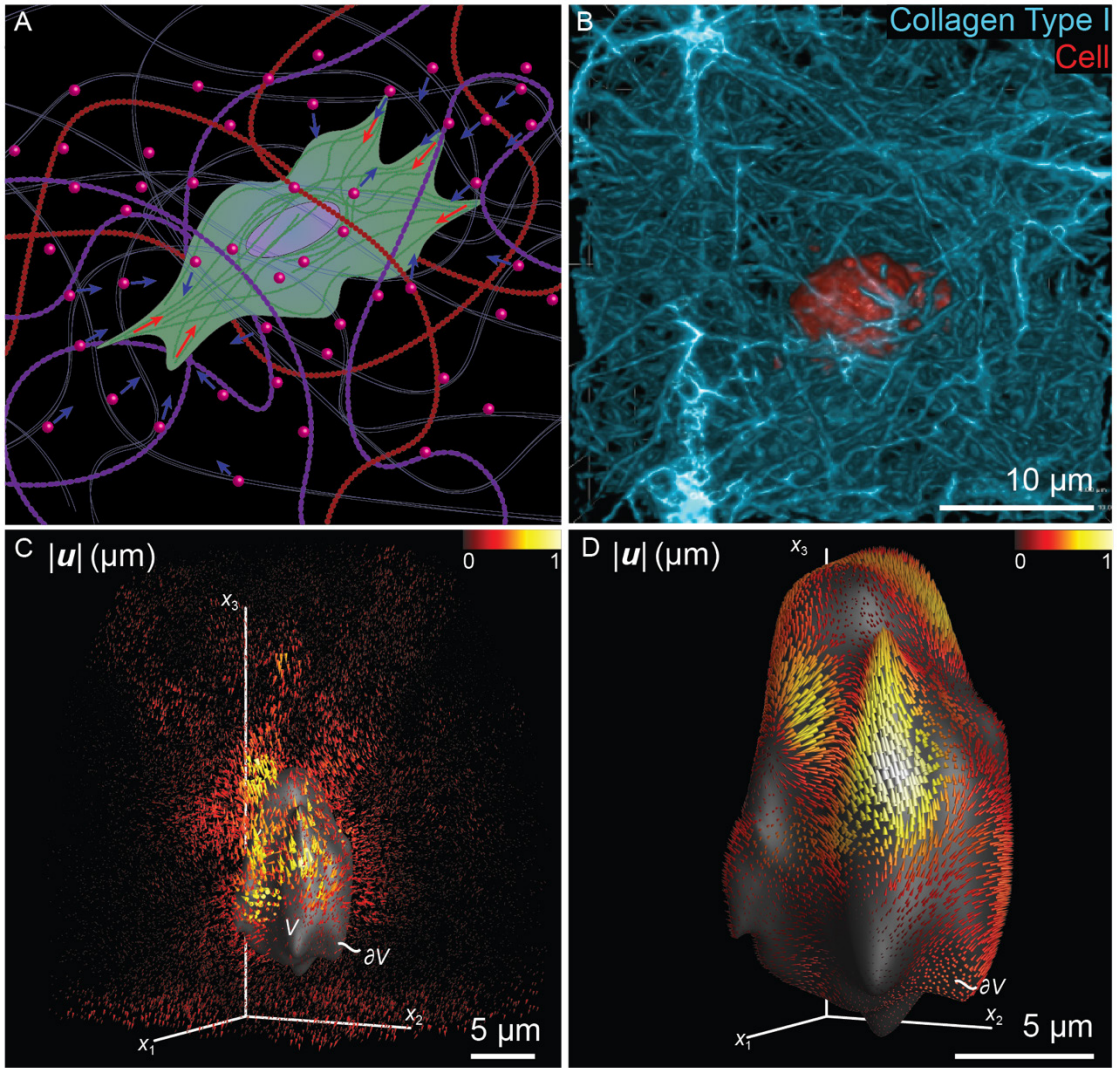
3D TFM. (A) Movement of marker beads in a 3D soft matrix are converted into an estimate of the 3D cellular displacements forces at adhesion sites. (B) A fully embedded human neutrophil (red) inside a collagen-I matrix (blue). (C) Image processing is used to track 3D matrix displacements. (D) 3D surface projections onto the cell surface for deduction of matrix displacements and strains. Cellular surface tractions can be deduced from (D) if matrix properties are known. Reproduced with permission from [120].

Around the year 2010, TFM started to explore new areas. The first studies that showed reconstructed traction forces not only for single cells, but also for cell ensembles of different sizes, from cell doublets to endothelial monolayers (‘monolayer stress microscopy’) [117]. The most important aspect of these studies was that cells could now balance their forces not only against the substrate, but also against neighboring cells. Around the same time, TFM leaped into the third dimension, both by reconstructing vertical cell forces on flat substrates (‘2.5 TFM’), and by considering cells fully embedded in three-dimensional hydrogels (‘3D TFM’) (figure 16) [118]. To date, some of the most physiologically advanced 3D TFM techniques feature collagen gels, which have important medical applications, such as wound healing or cancer metastasis. For cells in such 3D fibrous environments, methods were developed to either establish an appropriate material model [119] or to formulate a TFM-procedure that does not require a material law [120].

Based on these advances, at present, various TFM formulations exist for reconstructing cellular force fields from nanometer to millimeter length scales, using either closed-form analytical solutions for relatively simple material platforms, or finite element and hybrid formulations accounting for more complex material interactions. The main goal and intent of TFM is the correlation of physical forces with cellular, often structural, data, ranging from the size of individual adhesion sites through the spatial organization of the cytoskeleton, to the migration characteristics of a whole cell. These observations have spurred the development of hybridized advanced mathematical models that are capable of providing more detailed structural information than TFM experiments alone (‘model-based TFM’). Such approaches have been used to estimate the forces inside individual stress fibers via 2D TFM [121], and to understand how individual stress fibers can generate force through clutch-like mechanisms in a fibrous matrix, using 3D TFM [122]. Only through TFM has it become evident how important physical forces are for biological systems, e.g. for cell adhesion, migration, differentiation and fate (‘mechanobiology’).

#### Current and future challenges.

TFM has certainly excelled at providing high-resolution detail on cellular force generation in single cells in 2D, and more recently in 2D monolayers and 3D, but in order to keep pace with the ever-increasingly complex biological questions in mechanobiology, it now has to evolve along many different directions at the same time. As the biological applications become more demanding and sophisticated, opportunities exist to further refine the resolution and integration capabilities of TFM into quantitative and correlative biophysical frameworks. At the molecular level, we need increased integration with imaging and high throughput data. On the imaging side, this may comprise close integration with super-resolution and light sheet-based imaging techniques of cell adhesions, the cytoskeleton, the nucleus and the extracellular matrix, as well as fluorescent sensors reporting local activation of regulatory molecules (such as the small GTPases Rho and Rac) and forces on single molecules. Regarding high throughput advances, TFM has already been combined with RNA-interference in a high throughput manner, and it could also be combined with other high throughput techniques, such as mass spectrometry, CRISPR/CAS9 gene editing, or single cell sequencing. At the tissue level, we expect a move towards more medically relevant multicellular systems, e.g. clusters of cancer cells moving through fibrous tissue or organoids with specific functions grown from induced pluripotent stem cells. This will create interdisciplinary opportunities for biologists, physicists and engineers to work side-by-side to address these exciting new challenges. At the same time, however, this also creates the need for rigorous quantitative benchmarking and validation data sets, to evaluate and standardize the ever-growing arsenal of TFM and TFM-like methodologies that will emerge in the process. As it is common in bioinformatics and image processing, there is also a need to write and maintain publicly available TFM-software that can be used easily by a large user community. Finally, the handling and processing of large amounts of multispectral, spatial and time-lapse data will need to be addressed, especially for high-throughput or clinical investigations. The use of machine-learning and artificial intelligence algorithms may provide unique opportunities to link up with TFM, to address both multi-modal imaging and high-throughput data challenges.

#### Advances in science and technology to meet challenges.

Currently, a large effort has been invested in improving the procedures of classical TFM, using concepts and methods from physics, engineering and materials science [115–117]. First, one requires an accurate methodology for measuring cell-generated substrate displacement fields on a routine basis, but with high precision [120]. While there exist many popular choices of single particle tracking, particle image velocimetry and optical flow schemes, they might fail in the case of large deformations. A recurrent issue is also the need to track these deformations in relation to a stress-free reference configuration. Recently, a method has been developed to circumvent this need by printing a marker system onto the undeformed substrate (‘reference free TFM’) [123]. Next, we need to improve on the accuracy of our physical material (constitutive) description of the substrate from which the displacement and traction fields are deduced, both in 2D and 3D. Even to date, most TFM investigations have relied upon the use of polyacrylamide gels, which possess excellent idealized homogeneous and elastic material behaviour. Yet, as tissue culture models move more towards physiologically-realistic extracellular matrix design, or even into real tissue, TFM investigations will need to extend their own framework to account for more complex material descriptions [119]. This, in turn, will require the development of advanced experimental techniques for providing accurate descriptions of the local, as opposed to bulk, mechanical properties of the extracellular matrix surrounding the cells. Finally, an appropriate physical model for converting cellular displacements into surface tractions and forces is needed [120]. The development of tightly integrated experimental-theoretical frameworks [121, 122] will allow us to resolve more geometrically complex boundary value problems of cell–matrix and cell–cell interactions, e.g. clusters of cancer cells migrating and generating force in 3D.

Going beyond the classical formulation of TFM, the field is wide open for innovative approaches to reconstruct cellular forces from the deformation of a calibrated deformation sensor. For example, two interesting techniques have recently emerged to measure cell forces from podosomes, which are vertically organized adhesion sites used for invasion by immune, bone and cancer cells. In the first technique (‘protrusion force microscopy’), an atomic force microscope is used to measure podosome-generated bumps on a thin elastic membrane [124]. In the second technique (‘interference stress microscopy’), an optical interferometer is used to detect cell forces to the top surface of an elastic resonator cavity [125]. Both techniques do not require any measurement of a stress-free reference state and track deformations directly with very high resolution. These examples show that creative innovations can be used to fill gaps in the classical methodology, to answer very specific biological questions.

#### Concluding remarks.

While the original TFM methodologies have allowed us to appreciate the significance of physical forces in cell biology, more advanced formulations will be needed to keep pace with the rapid development in the life sciences, materials design, tissue engineering and imaging technologies. As biological investigations open up new frontiers in revealing complex biological behaviour, quantitative TFM approaches will be required to correlate the wealth of 2D and 3D fluorescent data to physical interactions inside, outside and among cells. This, in turn, provides a tremendous opportunity for experimental and modelling communities to come together and develop new methods, frameworks and models to quantify and correlate the biological world.

### Acknowledgments

U S S is a member of the cluster of excellence CellNetworks and of the Interdisciplinary Center for Scientific Computing (IWR) at Heidelberg.

## Super-resolved traction force microscopy

### H Colin-York^1^, C Eggeling^1,2,3^ and M Fritzsche^1,2^

^1^ MRC Human Immunology Unit, Weatherall Institute of Molecular Medicine, University of Oxford, Headley Way, OX3 9DS Oxford, United Kingdom

^2^ Kennedy Institute for Rheumatology, University of Oxford, Oxford OX3 7LF Oxford, United Kingdom

^3^ Institute of Applied Optics, Friedrich Schiller University, Jena, Germany

^4^ Leibniz Institute of Photonic Technology (IPHT), Jena, Germany

#### Status.

Super-resolved traction force microscopy (TFM) has the potential to transform live-cell mechanical force measurements [126, 127]. Conventional TFM is perhaps the most commonly used technique for mechanical probing in cells, owing to the simplicity of its implementation on most laser-scanning confocal microscopes. In TFM experiments, cells adhere to the protein functionalized surface of a thin (20–30 *µ*m) elastic gel [116]. Within the gel, immobilised fluorescent beads serve as fiducial markers, and imaging of the bead positions over time during the application of cellular tractions, allows the elastic displacement of the gel to be quantified. Combining the displacement measurements with the mechanical properties of the gel allows the forces applied by the cell to be recovered. The greatest shortcoming of TFM is the limited sensitivity due to the finite density of the fiducial markers, at which a displacement field can be sampled [126]. Consequently, the resolution limit of conventional fluorescent microscopes imposes a fundamental limit on the length scale of mechanical probing [128].

**Figure 17.**
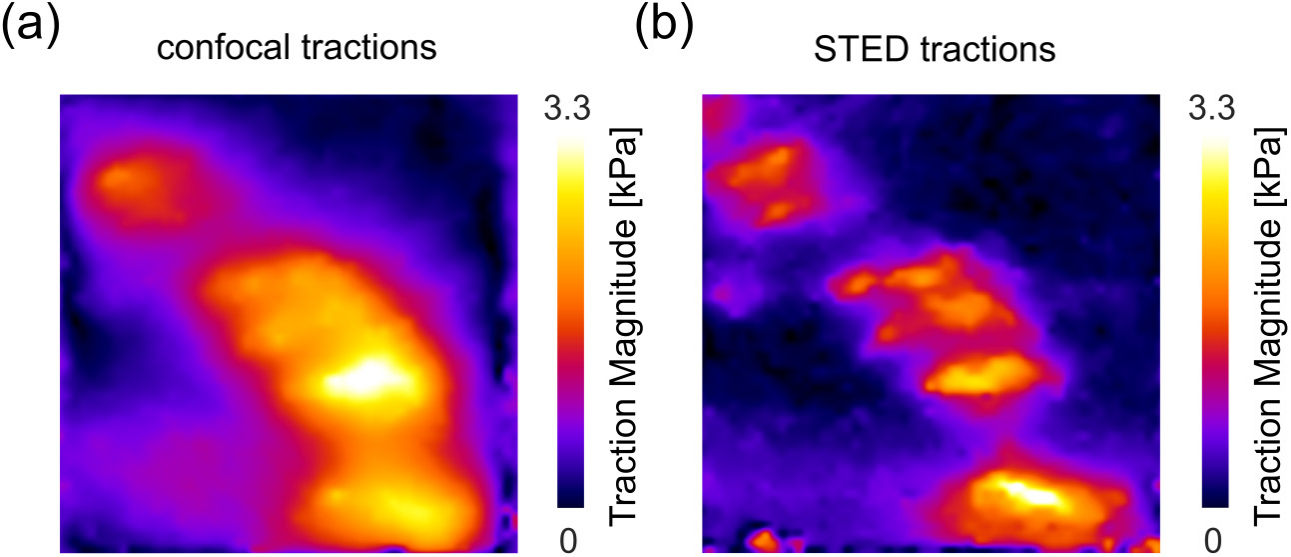
STFM applied to HeLa cell focal adhesions. (a), (b) Traction magnitude calculated from the measured confocal (a) and STED (b) recordings of the bead displacements. Scale bars, 2 *µ*m.

To overcome this challenge, we recently improved the spatial resolution and accuracy of TFM using super-resolution optical stimulated-emission-depletion (STED) instead of confocal or wide-field microscopy [126, 127]. The increased spatial resolution of super resolution TFM (STFM) allows a greater than five-fold higher sampling of the forces generated by the cell than conventional TFM, leading to more accurate quantification of cellular tractions (figure 17).

STFM naturally suffers from the restrictions inherent to all TFM and STED experiments, but it is likely to become a standard-tool for the quantifications of mechanical force production in living cells, because the resolution of STFM is theoretically not limited, as it scales with the applied STED power [129]. Ultimately, improving the spatio-temporal sampling of force measurements allows the quantification of mechanical force production in unprecedented detail, providing biomedical research with a state-of-the-art force probing technology to unravel the molecular mechanisms underlying mechanical force production in living cells.

#### Current and future challenges.

Several important challenges must be addressed to further improve the (S)TFM protocol. Advances can be achieved in the TFM and STED instrumentation, as well as in the fluorescent dyes of the fiducial markers.

The nature of the (S)TFM setup requires all imaging to be done at the top surface of the gel, meaning that imaging is subject to aberrations induced by the mismatch between the refractive index of the gel and that of the immersion medium [116, 126]. Improvements in aberration correction using, for instance, specialized objectives with refractive-index mismatch corrections, as well as using adaptive optics, would reduce the impact of these effects and would result in an improved spatial resolution, possibly along all three spatial dimensions, allowing even higher bead densities to be used and maximizing the accuracy of the TFM and STED instrumentation.

While the localization accuracy of STFM is theoretically not limited, for any given experiment, the sampling of the displacement field must meet the Nyquist criteria i.e. the spatial sampling frequency of the displacement field must be twice that of any feature that may be resolved in the displacement field [127]. This restricts the density of fiducial markers that must be high enough to reflect the complexity of the traction field that is applied by the cell. If the bead density is too low, areas of the gel will move without being reported by any bead movement, and the traction information will be lost. Conversely, if the bead density is too high, information will also be lost, as a result of the point spread functions of each individual bead overlapping with those nearby, making their relative displacements hard to recover accurately. Further, the maximal bead density within the gel must also be balanced with the ability to track the beads and the fluorescence light sensitivity of the biological specimen. Consequently, the quality and robustness of STFM experiments could be improved by better preparation of the gels with precise, regular, and pre-determined bead concentrations e.g. using substrate printing systems.

STFM precision is also affected by the acquisition duration of the fiducial marker positions. Only sufficiently fast acquisition times allow accurate tracking of the bead positions over time, which directly depends on the scanning speed of the microscope laser beam. Faster scanning beams, therefore, can further improve the quality of STFM experiments.

The resolution performance in super-resolution microscopy is profoundly related to the properties of the fluorescent dyes. Superior fiducial markers, using fluorescent dyes with improved photo-stability, switching performance and quantum efficiencies, reduce the light illumination of the cells and thus prolong the total time of STFM experiments. In this way, the accuracy and the total time period of STFM experiments, and thus the fluorescence light sensitivity of the biological specimen, can be improved by brighter and more stable fiducial markers.

The spatial resolution of STFM also depends on the reconstruction method used to recover the displacement of the beads from the fluorescent images [126, 127]. The most common methods of extracting bead displacements are those based on single particle tracking, where each individual bead must be localized, and those methods based on statistical comparisons of fluorescent images, such as particle image velocimetry (PIV). To this end, the image is divided into a grid, and each grid element is spatially correlated between frames to assess the degree of movement. PIV does not require localization of each bead but is limited spatially by the size of the grid elements required to give accurate correlations. Hence, the development of more advanced reconstitution algorithms could improve the quality and robustness of STFM experiments.

#### Advances in science and technology to meet challenges.

Emerging technical advances in optical super-resolution microscopy and the design of fluorescence dyes present the opportunity of establishing high-performance mechanical probing as a day-to-day toolbox, in the near future.

To this end, the recent development of 3D-STED [120] allows an even greater sampling for STFM of the forces perpendicular to the gel surface, in the same way that 2D-STED can increase the accuracy of the tangential force reconstruction. TFM has previously been successfully extended to measure 3D bead displacement, generated by cells within 3D elastic environments [120].

Fluorescent beads are bright and photo-stable, but are limited by photo-bleaching at high frame-rates or long acquisition times. Conventional beads could be replaced by custom-designed fluorescent nano-materials, such as nano-diamonds with luminescent properties, e.g. nitrogen-vacancy centers that possess good bio-compatibility, high photo-stability, and facile surface functionality [130].

Acquisition of the fiducial markers is limited by the recording duration of the observation region, which could be improved by parallel scanning of multiple regions of interests, or by wide-field based illumination of the complete field of view through, for instance, structured illumination [131].

#### Concluding remarks.

STFM imaging is established as a valuable tool for biological research and its application must be extended to a wider range of scientific questions. The development of STFM will involve the implementation of improved fluorescent dyes with increased photo-stability, allowing a reduction in photo-toxicity. In addition, new microscope technologies will be necessary, as extensions to more complex biological specimens will benefit from further improvements in the spatial and temporal resolution, as well as the ability to image thicker specimens. Combining STFM with high-performance objectives for deep-substrate imaging and/or the application of adaptive optics, holds a significant potential benefit for mechanical probing in immunology, biophysics, and other areas. Further, the increasingly improved usability and availability of super-resolution systems will reduce the need for external technical support, thus facilitating the potential of STFM to become the standard tool for mechanical force probing in the field of biomedical research.

### Acknowledgments

We greatly acknowledge support from the Wolfson Imaging Centre and funding by the Medical Research Council (grant No. MC_UU_12010/unit programme G0902418 and MC_UU_12025), MRC/BBSRC/EPSRC (grant No. MR/K01577X/1), Wellcome Trust (grant reference 104924/14/Z/14 and Strategic Award 091911 (Micron)), Wolfson Foundation, and Oxford internal funding (EPA Cephalosporin Fund, John Fell Fund).

## Electrophysiology on lipid bilayers combined with fluorescence imaging and spectroscopy

### Richard Wagner^1^, Satya Prathyusha Bhamidimarri^1^, Niklas Brending^2^ and Mathias Winterhalter^1^

^1^ Department of Life Sciences & Chemistry, Jacobs University, Bremen, Germany

^2^ Ionovation GmbH, Osnabrück, Germany

#### Status.

Classical planar lipid bilayers containing reconstituted ion channels, transporters and pumps have already served for long time as a well-defined model system for high-resolution electrophysiological investigations of ion channel and large pore forming solute channels (porins) functions. More recently, suitably designed microchips containing free-standing horizontally oriented bilayers (HLBs), allow the combination of electrophysiology with high-resolution nanoscopic fluorescence techniques down to the single-molecule level. Combining simultaneous single-molecule fluorescence measurements of membrane protein conformational changes with high-resolution electrophysiology, has been generally regarded as a promising tool to gain insights into the structure–function relationships of ion channels at the single molecule level [135]. As proposed almost 20 years ago, the holy grail of membrane channel studies is to produce an atomic scale movie of an ion channel or large porin-like channel solute at work, simultaneously observing conformational and electrical or solute property changes, as ions or uncharged solutes flow through the protein [136]. Regardless of the early identification of this challenge, one has to admit that up to now only a few publications employing directly correlated simultaneous fluorescence and electrical recordings on single ion channels appeared in high-ranking journals [134]. HLBs, in combination with nanoscopic fluorescence techniques, were also successfully employed to probe physico-chemical properties of bilayers and the topology of membrane proteins [134, 137]. Obtaining a molecular movie of a single transporter slow turnover action would require HLBs containing these transporters, while solely using nanoscopic fluorescence microscopy [28] to monitor transport and simultaneous changes in the protein. One attempt to follow membrane transport of uncharged solutes at a single molecule level in HLBs, is the optical single transporter recording approach, using confocal microscopy and HLB-arrays [138]. The combination of high-resolution fluorescence and electrical recording allows the monitoring of the transition of water soluble toxins, which form high conducting membrane pores upon transition from solution into the membrane [132]. Correspondingly, it should be possible to monitor the transition of water soluble receptor-cargo complexes into the membrane, thereby forming membrane pores [139]. Finally, fluorescent ligand binding combined with single-channel recording, is aimed at providing mechanistic insight into the behavior of ligand-gated channels [28].

**Figure 18.**
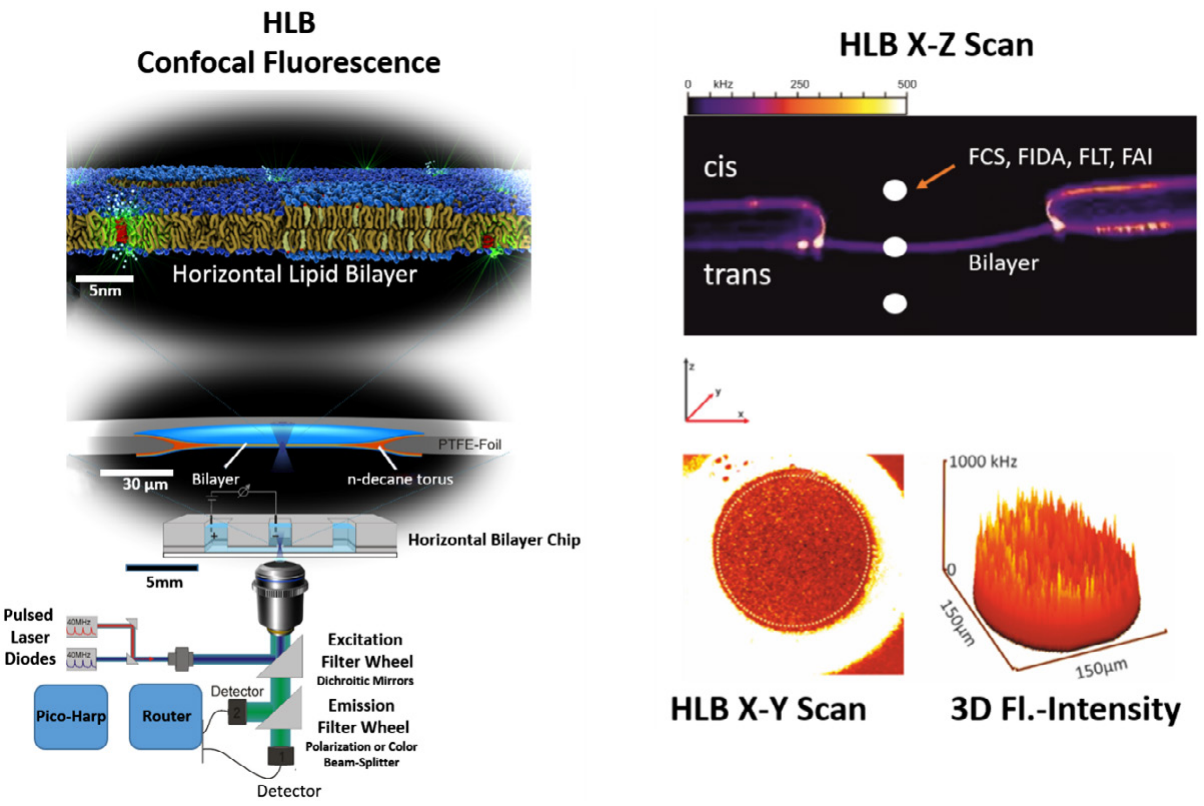
Principal setup of a horizontal lipid bilayer (HLB) combined with a confocal scanning spectrometer allowing high resolution fluorescence imaging and spectroscopy [132–134].

#### Current and future challenges.

Single molecule methods provide a unique insight into the function of biological systems, compared to macroscopic average values. They are primarily able to measure the distribution of the biomolecule behaviour or properties and not just their ensemble mean values. As a robust *in vitro* system, artificial bilayers can reproduce the full range physico-chemical properties of the biological membranes, and are thus an adequate environment for the functional reconstitution of membrane proteins. The expansion of the classical bilayer technique, where ion channels are analysed by electrical single channel measurements, in combination with the nanoscopic fluorescence techniques (microscopy/spectroscopy) in HLBs, decisively extends the possibilities of this *in vitro*-system. (i) Free standing HLBS and partially supported HLBs in combination with high-resolution fluorescence techniques allow to examine;

(i) the properties of lipid mixtures forming the bilayer membrane.
(ii) the topology and oligomeric states of membrane proteins
(iii) the transition of water-soluble proteins forming membrane pores upon transition into the membranes (pore-forming toxins, receptor/cargo complexes).
(iv) Also, ligand gating of ion channels can be investigated using fluorescent effectors, and electrical single channel recording can give molecular details on ligand binding affinities and how binding is transmitted to channel gating.
(v) Low rate transport of uncharged metabolite solutes by large pore forming channels like the classical bacterial porins can be investigated using electrical and confocal fluorescence recording with HLBs.

**Figure 19.**
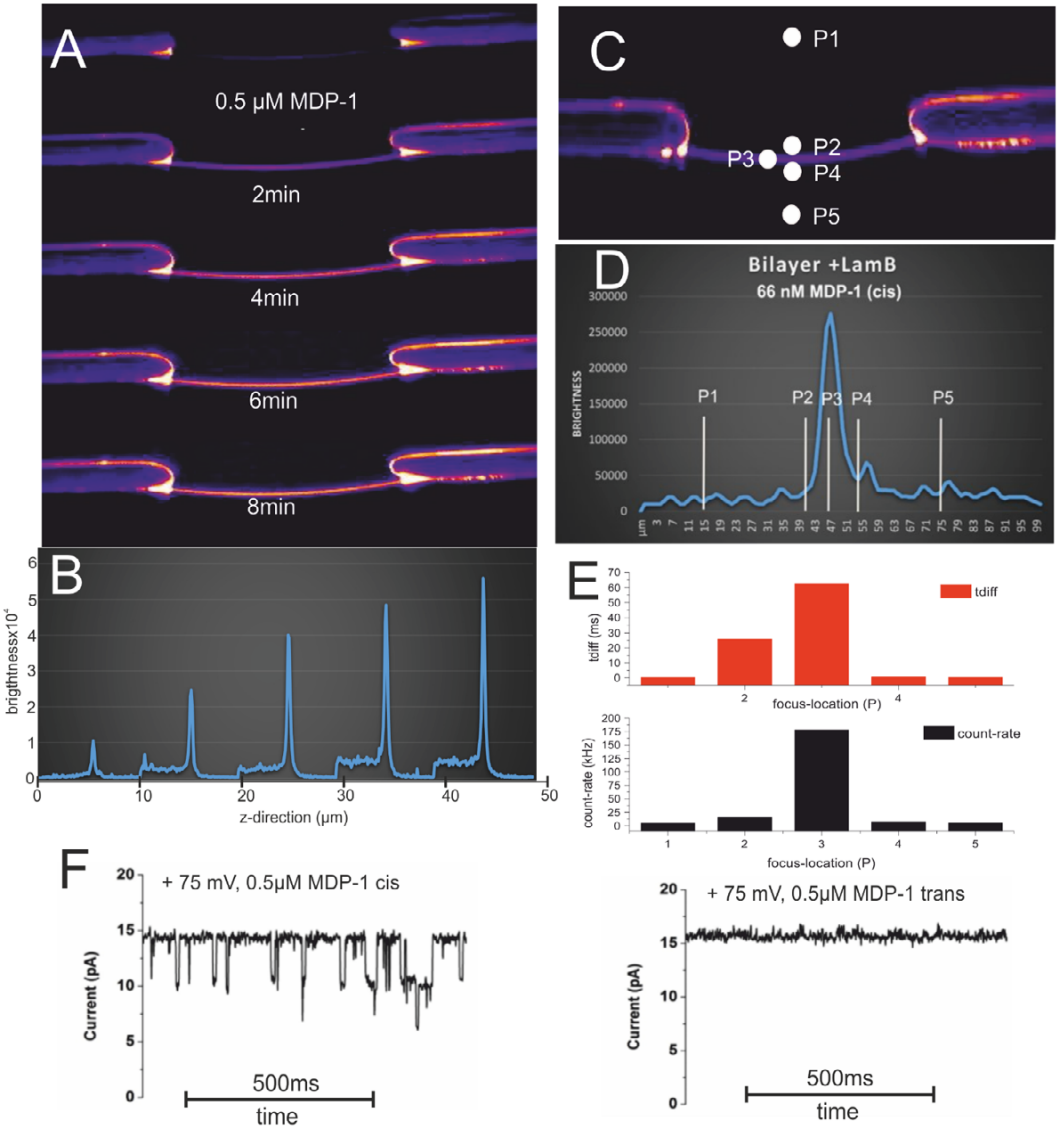
(A) shows a stack of confocal *x*-*z* scans through a LamB containing bilayer (POPC/POPE/POPS 8:1:1) after addition of 60 nM MDP-1 (cis side, buffer symmetrical (cis/trans) 1 M KCl 10 mM HEPES pH 7.2) at *t*  =  0 recorded with time intervals of Δ*t*  =  2 min for each following *x*-*z* scan (total distance per scan 100 *µ*m). (B) Recorded fluorescence intensity along the HLB *z*-coordinate. (B) Corresponding molecular brightness along the *z*-direction. (C)–(E) FCS measurements along the HLB *z*-coordinate. (F) Electrical single channel recording from a bilayer revealing that MDP-1 induces channel blocking only from the cis site. As shown by sequential confocal *z*-scans of the HLB MDP-1 when added to the cis HLB-compartment binds in a time dependent course to the bilayer (A) and (B) and remained stably bound to the membrane after repeated careful perfusion of the cis compartment (not shown). Intensity profile along the *z*-direction of the HLB (D) indicates that MDP-1 is stable bound to LamB in the bilayer which was confirmed by competition with non-labelled MDP which released the bound MDP-1 from the membrane (not shown).

One recent example for this is given in figure 19, which shows the HLB containing multiple copies of LamB, a substrate specific *E. coli* channel protein, which contains specific maltose binding sites and is part of the bacteria-specific maltodextrin transport pathway. We investigated the specific binding of a fluorescent MDP composed of maltohexaose, carrying a perylene fluorophore (MDP-1). Through fluorescence correlation spectroscopy (FCS) measurements along different points of the *z*-axis, we could characterize three different mobility classes of MDP-1, with ${{\tau }_{(dif)}}\cong 0.2\,{\rm ms}$ (P1) in solution to ${{\tau }_{(dif)}}\cong 25\,{\rm ms}$ (P2) close to the bilayer and ${{\tau }_{(dif)}}\cong 62\,{\rm ms}$ at the bilayer bound to LamB (see figure 19(C)). The results show also that MDP-1 is not permeable through LamB.

#### Concluding remarks.

HLBs, in combination with the single-molecule techniques of the electrical single channel recording and nanoscopic fluorescence microscopy/spectroscopy, offer great potential with regard to their ability to resolve biological processes *in vitro* down to the molecular level of single steps. Despite this, few publications on the use of these combined techniques are found in the literature. In our opinion, this is mainly due to the high technical requirements of the individual disciplines. So far only very few robust HLB-systems with possible access to the solution on both sides are commercially available. In order to facilitate the combined technique, we believe it is necessary to develop fully integrated HLB systems in which arrays of bilayers are automatically generated. These array chips with automated liquid handling must be individually, electrically addressable. Such an automatic HLB system, transferred into a suitable environment with state of the art measuring technology for nanoscopic fluorescence techniques (microscopy/spectroscopy) and electrical single/multichannel techniques, could develop the full potential of the technology.

### Acknowledgments

The research was conducted as part of the TRANSLOCATION consortium and has received support from the Innovative Medicines Initiatives Joint Undertaking under Grant Agreement No. 115525, resources that are composed of financial contribution from the European Union’s seventh framework program (FP7/2007–2013) and EFPIA companies in kind contribution.

## Voltage-clamp and patch-clamp fluorometry: studying ion channels and transporters with light

### Jana Kusch^1^ and Giovanni Zifarelli^2^

^1^ University Hospital Jena, Jena, Germany

^2^ Department of Physiology, Anatomy and Genetics, University of Oxford, Oxford, United Kingdom

#### Status.

To gain a deep understanding of the complex dynamic behavior of proteins, structural and functional data need to be integrated. For ion channels and ion transporters, functional information can be collected with an extraordinary high time-resolution by voltage-clamp and patch-clamp techniques. These approaches, with their numerous variations, revolutionized ion channel/transporter research significantly. Nevertheless, conclusions about structural rearrangements within a protein, dynamic protein interactions or changes in protein mobility, can only be drawn indirectly. Structural methods, like x-ray crystallography, nuclear magnetic resonance or cryo-electron microscopy, on the other hand, produce a wealth of structural information, with a resolution down to the atomic level. However, these approaches provide only static snapshots of highly dynamic processes and in most cases, the functional state associated with a given structure is not even fully defined.

**Figure 20.**
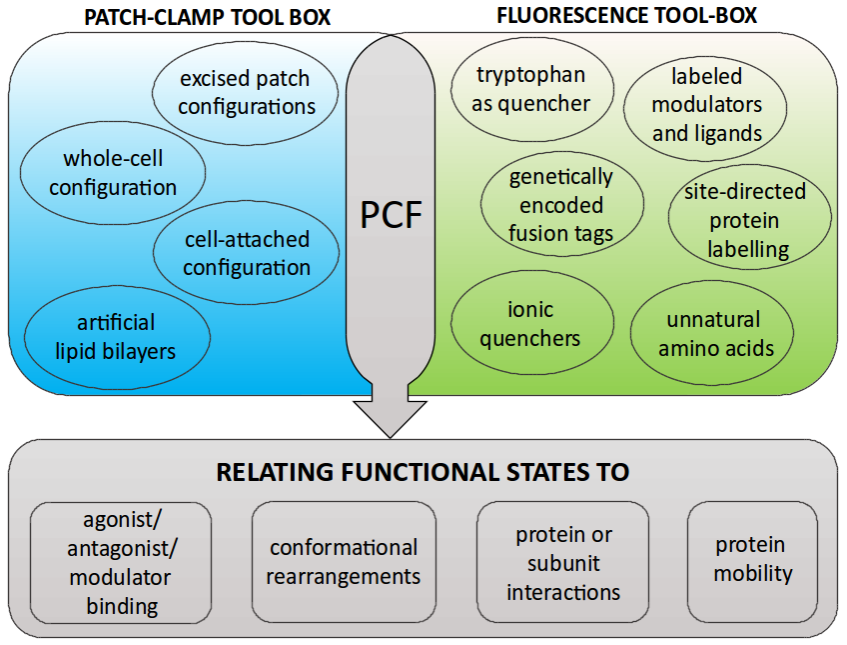
Overview of the main electrophysiological and spectroscopic tools combined in PCF. The high variability of PCF, given by the number of possible combinations of electrophysiological and spectroscopic tools, makes it a potent approach for studying ion channels, thus overcoming the limitations of the individual techniques. Reprinted from [143], Copyright 2014, with permission from Elsevier.

To overcome these limitations, classical electrophysiological approaches were combined with different kinds of spectroscopic techniques. Thus, ion channel/transporter function can be monitored simultaneously with conformational changes, changes in protein or subunit interactions, ligand binding events, or protein mobility, giving the possibility to directly correlate these functional and structural/spatial parameters. The first step in this direction was made in the 1990s, through the establishment of the so-called voltage-clamp fluorometry (VCF), that employed either the two-electrode voltage clamp [140] or the cut-open technique [141] to investigate ion channels in whole *Xenopus laevis* oocytes. Typically, in VCF, labeling of the protein is carried out by site-directed sulfhydryl-modification of cysteine residues, but more and more frequently by fluorescent unnatural amino acids. Inspired by the principles of VCF, several types of patch-clamp fluorometry (PCF) approaches were designed, combining different electrophysiological and spectroscopic tools, currently only for ion channels [142]. The various components reported so far are summarized in figure 20 [143].

Despite the results already achieved, both VCF and PCF are still under continuous development. Whereas up to now, mostly *in vitro* experiments on protein ensembles were performed, further advances might set additional foci on single-channel and *in vivo* approaches.

**Figure 21.**
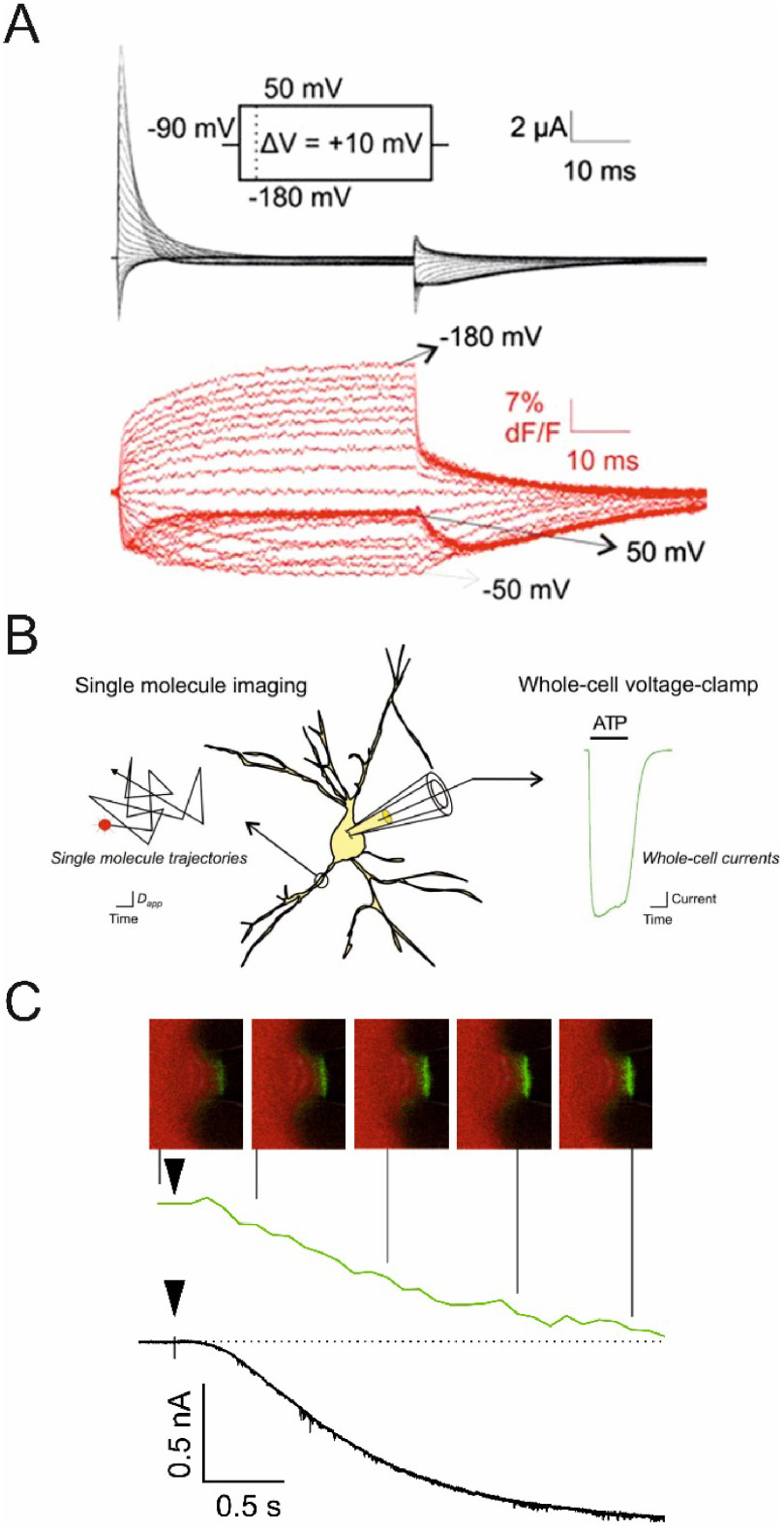
Three examples illustrating the wide variety of combining electrophysiological and spectroscopic approaches. (A) VCF with *Xenopus laevis* oocytes expressing voltage-dependent potassium channels (K_V_1.2/2.1 chimera). Fluorescence signals arising from the incorporated unnatural amino acid Anap (red traces) and gating currents (black traces) were recorded in response to depolarizing voltage jumps. Reproduced with permission from [145]. (B) PCF combining single-particle tracking and whole-cell patch clamp. Neurons expressing P2X2FLAG–YFP receptors were labeled with QDs and imaged over time with epifluorescence optics (black trajectories). At the same time, whole-cell voltage-clamp electrophysiology (green trace) was used to measure transmembrane currents before, during and after ATP applications. Reproduced with permission from [146]. (C) PCF combining confocal laser scanning microscopy and inside-out patch-clamp configuration. Patches were excised from *Xenopus laevis* oocytes expressing mHCN2 channels. Fluorescently labeled cAMP (fcAMP) was applied to study ligand binding (green trace) and channel activation (black trace) in response to an activating hyperpolarizing voltage jump (black triangle). Reprinted from [147], Copyright 2010, with permission from Elsevier.

#### Current and future challenges.

The core of VCF/PCF approaches, is to correlate two sets of different types of data (ion current and fluorescence changes), which are collected using different types of recording devices in the same experiment. In this context, a major challenge in most of the PCF approaches is to ensure that the protein population, which contributes to the spectroscopic response, is identical to the population contributing to the electrophysiological response. However, in practice there might be unlabeled proteins contributing to the current and labeled proteins being electrically silent. The second precondition for a useful correlation is to perfectly synchronize spectroscopic and electrophysiological data.

As in other live-cell imaging approaches, several sources of background fluorescence can disturb spectroscopic recordings. One major source is autofluorescence, the natural emission of light by biological components. Except for approaches in which genetically encoded fluorophores are used, background fluorescence can additionally arise from (1) unspecific binding of fluorophores to molecules nearby the proteins of interest, (2) unspecific binding to equipment surfaces like patch pipettes, and (3) unbound fluorophores in the surrounding bath solution. Importantly, negative control recordings to determine background intensity have to be performed under voltage-clamped conditions, because fluorophore binding to proteins and surfaces might be voltage-dependent.

However, one of the biggest challenges in VCF/PCF, as in live-cell imaging in general, is to find a fluorophore whose physical and chemical features match the experimental needs, and which does not affect the protein behavior when being attached to it. To this aim, it is required to (1) identify the most suitable probe (excitation and emission wavelength, size and chemical structure, pH dependence, environment dependence, unspecific binding), (2) check for the optimal position in the protein, (3) check for the best tagging strategy (post translational labeling, genetically encoded protein dyes, fluorescent unnatural amino acids), and (4) take into account size and chemical structure of linkers between fluorophores and proteins.

#### Advances in science and technology to meet challenges.

As mentioned before, VCF and PCF are still under continuous development, particularly with respect to their spectroscopic components. Advances in both labeling and microscopy technologies are required.

Regarding labeling technologies, we expect that the rapidly progressing developments in the field of channel/transporter research will demand a parallel development of new and rather custom-tailored fluorophores. Like for other live cell imaging approaches, the challenge is to develop fluorescent molecules which are not only small, bright and photo-stable, but which also have a low cellular toxicity, offer a high signal-to-noise ratio, and do not interfere with the proteins of interest. For post-translational labeling, fast labeling kinetics and, in the case of intracellular targets, good membrane permeability is desired. Probe selection and design can be simplified because there is only a small number of ‘core’ fluorophores giving rise to a large ensemble of fluorescent probes by attaching various reacting groups, substrate moieties, chelating components, and other chemical entities to these core molecules [144].

This continuous development of new fluorophores and the increasing variety of biological systems used in PCF require a paralleled development of gentle chemical strategies, to mask unspecific binding sites in the sample. Preventing unspecific binding to equipment surfaces like glass substrates, demands new passivating materials which do not interfere with optical transmission or cell attachment.

To tackle the problem of autofluorescence, the strategy of choice is avoidance, demanding advances in labeling and microscopy technologies, which go hand in hand. This should provide better fluorophores with long excitation wavelengths, as well as optimized excitation and emission filters, and allow for improved dual wavelength correction or linear unmixing settings.

In VCF/PCF, as in fluorescence microscopy in general, there is an inherent trade-off between spatial resolution, time resolution and phototoxicity. Despite huge progress in the development of high- and super-resolution microscopy over the last years, further efforts are needed to get rid of sample fixation, paving the way for monitoring highly dynamic processes of proteins or protein domains without motion-induced blur. On the part of microscopical components, further optimization of objective lenses, filter sets, and detectors will help to acquire high resolution images without cell or protein damaging.

#### Concluding remarks.

Over the last two decades, VCF and PCF have been shown to be tremendously powerful approaches to shed light on the dynamic behavior of ion channel proteins. Essentially, there seems to be no limit to the imaginable combinations of spectroscopic and electrophysiological tools (figure 21). However, with the exception of a few studies dealing with single-channel recordings, up to now, the focus was clearly on the investigation of protein ensembles *in vitro*, monitoring a large number of channels, often several hundreds or thousands at the same time. Further efforts are expected to establish the technical conditions to facilitate more single-channel and *in vivo* experiments.

So far, in contrast to VCF, PCF has not been applied to ion transporters, most probably because of relatively low current amplitudes, particularly in cell patches. New strategies to increase protein expression levels and new recording devices with higher sensitivity might expand the applicability of PCF to transporter proteins in future.

### Acknowledgments

We thank Vasilica Nache for proofreading and commenting. Funded by the Deutsche Forschungsgemeinschaft (DFG, German Research Foundation)—TRR 166 project B7 to J K.

## Fluorescence and magnetic resonance imaging

### Nyoman D Kurniawan, Viha Parekh, Michael W Vogel and David C Reutens

Centre for Advanced Imaging, The University of Queensland, Brisbane, QLD 4072, Australia

#### Status.

Preclinical studies using *in vivo* magnetic resonance imaging (MRI) often require downstream *ex vivo* histology and fluorescence imaging (FI). FI microscopy is vital for the validation of the underlying cellular changes observed by MRI. Currently, MRI and FI are rarely performed simultaneously, due to issues relating to optical hardware sensitivity and compatibility within the magnetic field, differences in sample preparations and temporal/spatial resolutions.

FI can utilise a vast array of biomarkers to study distinct cellular processes, and discrete information can be obtained from the same sample using reporter fluorophore molecules. Depending on the sensitivity of the optics, FI can capture images from micrometre to millimetre resolutions in single cells and tissue slices *in vitro* [148] and in live animals [149]. Ultra-high resolution FI is mostly performed with *in vitro* samples due to constraints on fluorescence labelling, optical magnification, depth of penetration and light scattering.

MRI detects the resonance radio frequency (RF) signals from nuclear spins inside a strong magnetic field, and is routinely used to obtain high-resolution images from intact living animals. The ability of MRI to image at sub-cellular resolution, its temporal resolution and the range of available MRI biomarkers, are more limited compared to FI. However, MRI has the advantage of yielding excellent tissue contrast without external labelling to measure biological structure and function, for example using proton spin relaxation and microscopic water diffusion [150].

#### Current and future challenges.

Conventional brain functional MRI (fMRI) indirectly detects neuronal activation by measuring the hemodynamic response given by T2^*^ dependent blood oxygenation-dependent (BOLD) signals. BOLD fMRI contains some temporal delays because the hemodynamic response is delayed by mechanisms underlying neurovascular coupling. In addition, BOLD fMRI may depict a larger area of brain activation as the signals can also originate from veins and capillaries that are distal from the site of neuronal activation [151]. Therefore, the development of new fMRI methods that directly detect neuronal activation are necessary to accurately map brain activity.

Neuronal activation involves membrane depolarisation with the initial influx of sodium ions, calcium ions and water being followed by the release of potassium ions into the extracellular space. The transient increase in intracellular water results in cell swelling, in which the proportion of slow water diffusion inside the cells increases, compared to the fast water diffusion outside the cells. Detection of such activity-dependent swelling has been proposed as a means of direct detection of neuronal activity using diffusion fMRI (dfMRI) [152].

Detection of pure dfMRI signals in living animals has been difficult. To achieve adequate temporal resolution, both dfMRI and BOLD fMRI utilise the EPI readout sequence. The specificity of dfMRI signals has been intensely debated, as they are suspected to be contaminated by the T2^*^ BOLD signal [153]. A key step in validation is to determine the specificity of dfMRI in a system that is free from blood flow interference.

**Figure 22.**
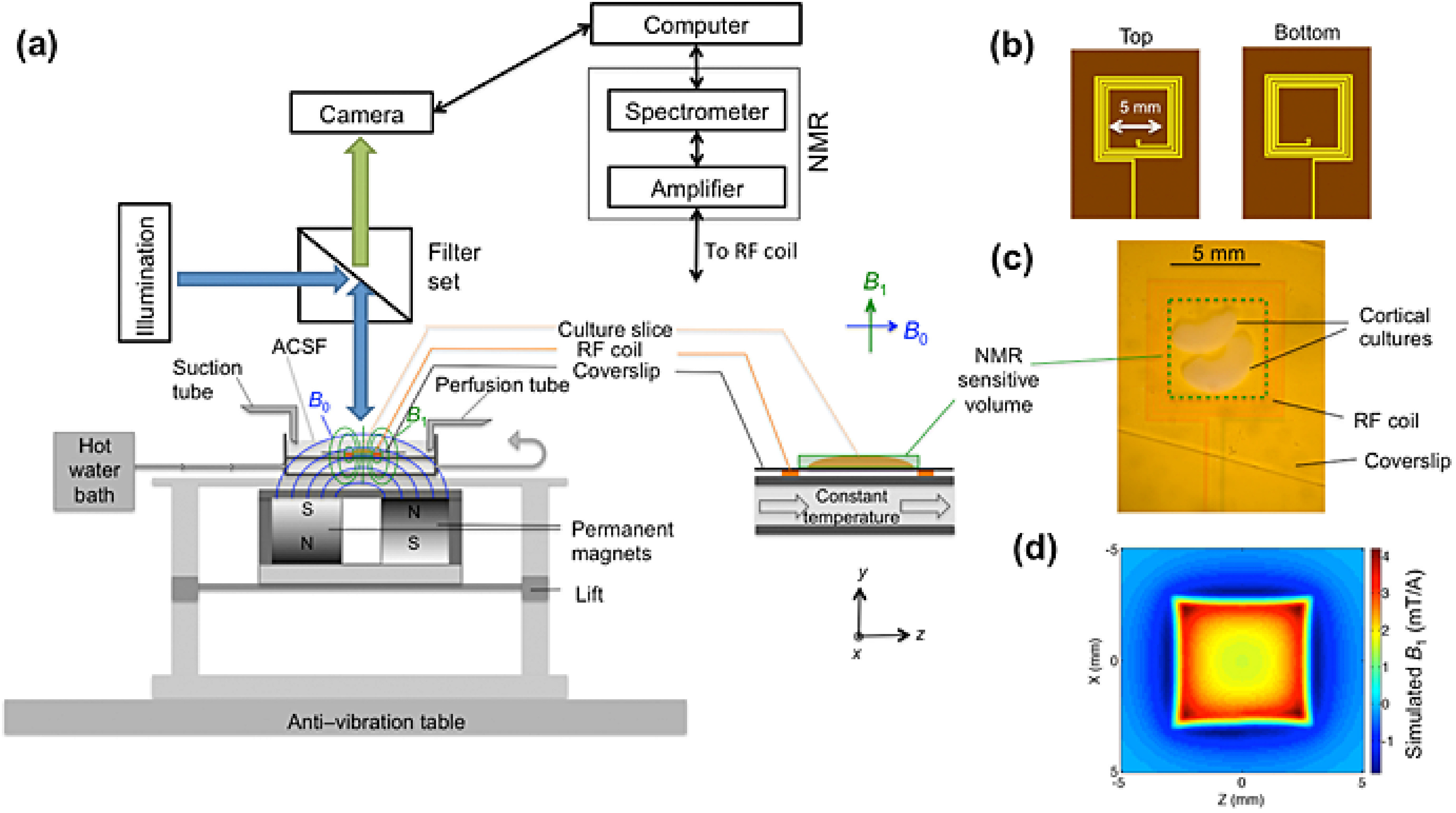
A platform for simultaneous *in vitro* fMRI and FI. (a) This system is based on an open MRI at 0.32 T (bottom table), a brain slice chamber with constant nutrient flow (middle) and a camera (top) for detection of fluorescence. (b) Two surface coils placed below and over the slices, (c) microscope image of the brain slice, (d) simulated excitation profile of the coils. [155] John Wiley & Sons. Copyright © 2015 John Wiley & Sons, Ltd.

Neuronal activation has been routinely measured *in vitro* using brain slices and calcium-sensitive fluorophores, to detect the influx of calcium ions [154]. Therefore, a hybrid imaging system that enables simultaneous *in vitro* brain slice studies using MRI and a calcium fluorophore can be used to validate dfMRI. A hybrid MRI and FI system (figure 22) was recently built using a single-sided MRI at 0.32 Tesla (T) to test dfMRI in organotypic cortical cultures [155]. The low magnetic field and the use of an RF surface coil provided the benefit of an open space above the magnet to accommodate a chamber with access for sample manipulation, a continuous perfusion system to control the sample physiological state, and the placement of optical hardware close to the sample for efficient detection of the fluorescence signals. This system, however, only produced MR signals (not MR images) so it could not be used to investigate spatial resolution of the diffusion signal. The use of a low-field magnet may also limit the sensitivity of detection of the diffusion signal.

#### Advances in science and technology to meet challenges.

New hardware advances in MRI involving the development of ultra-high field magnets (17 T for preclinical MRI), parallel imaging, cryoprobes and field gradients (>1 T m^−1^) are important to increase image sensitivity and resolution. Powerful gradient coils are important to obtain strong diffusion gradients within a short echo-time and to minimise artefacts in high field MRI. Parallel imaging and cryoprobes, however, will be difficult to implement in a hybrid system with FI, due to spatial constraints within the bore of the MRI.

New advances in FI involve the development of *in vivo* two-photon microscopy (TPM) [156] and ultra-sensitive photomultiplier (PMT) charged-coupled devices, which are useful to detect fluorescence signals from a single cell. New calcium-fluorescence indicators with no resting signal (e.g. Fluo4-AM) can be used to further enhance fluorescence detection upon calcium binding.

**Figure 23.**
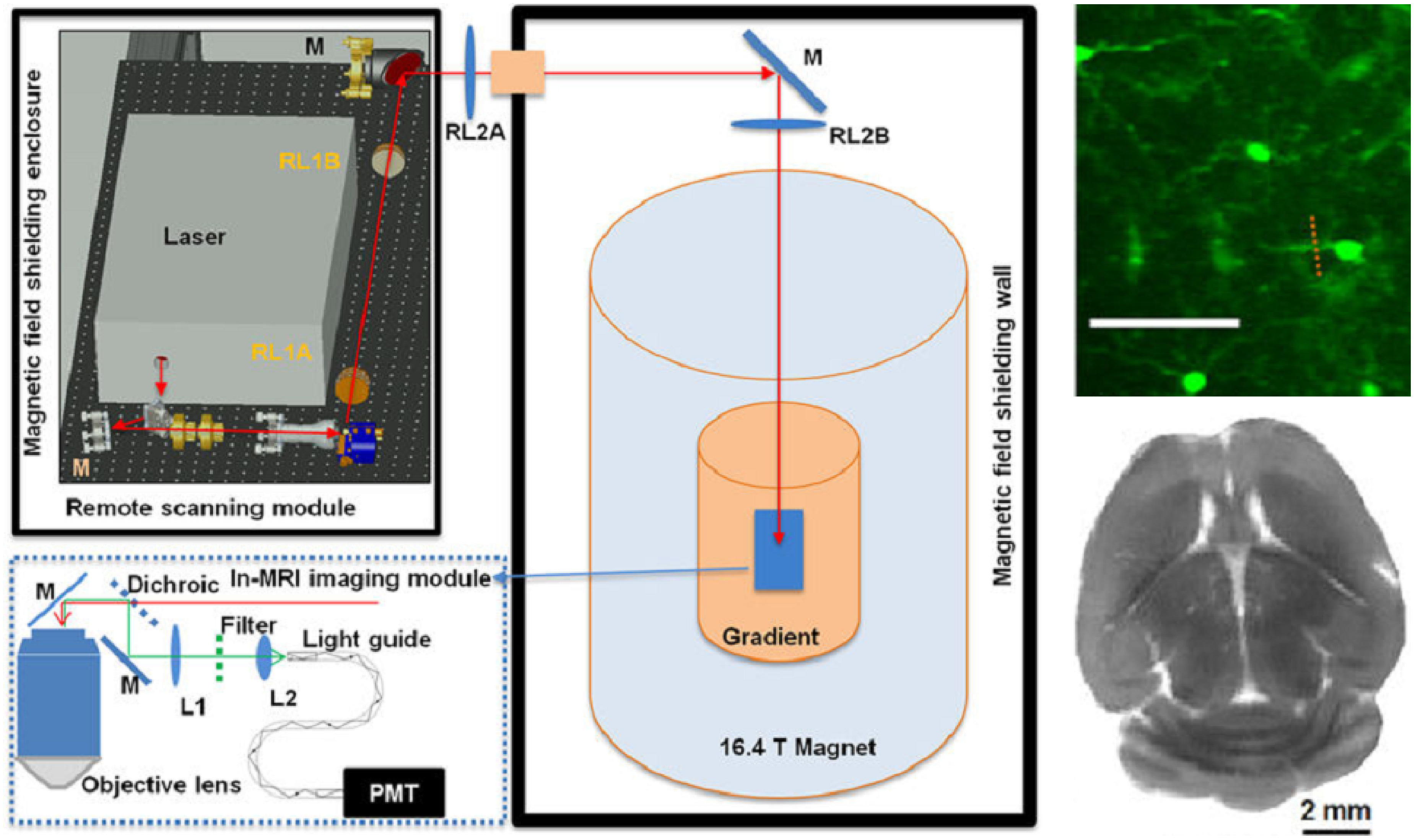
A proof-of-concept *ex vivo* platform for TPM and MRI at 16.4 T. The laser scanning module (upper left corner) was located outside the 16.4 T magnet room and the excitation laser beam was guided into the in-MRI imaging module (centre image) using fibreoptics. Simultaneous view of whole mouse brain using TPM (top right corner) and MRI (bottom right corner). Reproduced from [157] CC BY 4.0.

A proof-of concept hybrid platform for simultaneous imaging of a whole *ex vivo* mouse brain using TPM and MRI has recently been built at 16.4 T [157]. Key aspects of this system are the protection of the TPM module from the strong magnetic field, where laser excitation was directed to the sample via a set of relay lenses and mirrors. The fluorescence emission was relayed to the PMT module using a long light guide cable (figure 23). The TPM module produced ultra-high resolution images at a cellular level, but only within a small field-of-view; this was complemented by high-resolution images of the whole brain using MRI. Unlike a hybrid system using a single-sided low-field magnet, the space within the bore of the ultra-high field magnet is typically very limited. Therefore, there may be challenges in accommodating a chamber for live brain slices and apparatus necessary to achieve a constant flow of nutrients, and obtaining images in the absence of flow artefacts.

#### Concluding remarks.

The development of the platform for simultaneous MRI and FI acquisitions are still in its infancy. This is an exciting multi-disciplinary field of research requiring expertise in MRI, optical FI, instrumentation and neuroscience. The development of such hybrid systems will be significant not only for the validation of dfMRI, but also for providing a platform to understand the basis of MRI signals change at the cellular level.

### Acknowledgments

Mr Donald Maillet for engineering expertise.

## Fluorescence microscopy and scanning small-angle x-ray scattering: imaging of biological cells

### Florian Rehfeldt^1^ and Tim Salditt^2^

^1^ University of Göttingen, Third Institute of Physics—Biophysics, 37077, Göttingen, Germany

^2^ University of Göttingen, Institute for X-ray Physics, 37077 Göttingen, Germany

#### Status.

Fluorescence microscopy has enabled a massive gain in knowledge about the localization of proteins in cells, and thereby of intracellular structures, based on selective labeling using antibodies. Over the last two decades, super-resolution fluorescence microscopy has helped to replace the Abbe resolution limit by the size of the fluorescent label, as the ultimate resolution limit. While nanobodies, Fab fragments, and similar approaches, have significantly decreased this size, all these approaches are still relying on external labeling of the sample [28]. To overcome both the limit of label size and the constraints associated with only detecting the labeled entities, complementary techniques are required. To this end, small angle x-ray scattering (SAXS) is well known to resolve soft matter and biomolecular structure in unlabeled biomolecular solutions and suspensions, with a resolution down to below 1 nm, as limited by the largest scattering angle. While in its conventional form it is limited to ensemble averages, SAXS can be extended to offer real-space resolution and hence, a localization of the diffracting structures by combination with focusing optics [158].

**Figure 24.**
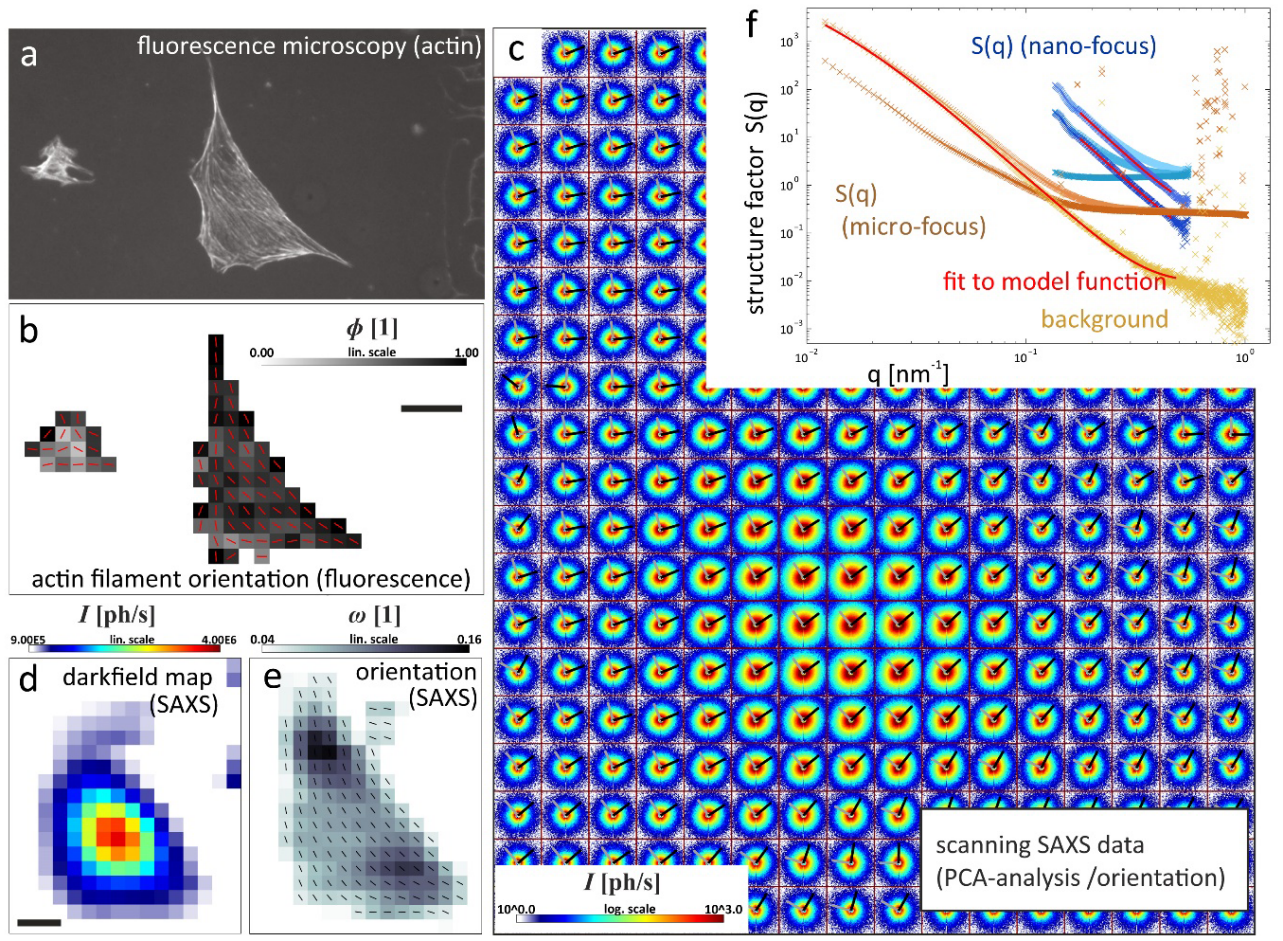
Scanning x-ray microscopy using a micro- or nano-focused beam. (a) Fluorescence microscopy shows the actin fiber distribution that is pixelwise analysed in (b). (c) Composite image of the scan area including the two rescaled eigenvectors (gray and black) from PCA. (d) Total SAXS signal provides an x-ray darkfield map and (e) anisotropy from PCA for each pixel. (f) Radial intensity profiles can be fitted to get more information in terms of form and structure factors. Reproduced from [163]. © 2017 IOP Publishing Ltd and Deutsche Physikalische Gesellschaft. CC BY 3.0.

Until recently, scanning SAXS was exclusively used for strongly scattering biomaterials such as bone or wood, and mostly with moderate x-ray focal spot sizes of several *µ*m. Progress in x-ray optics and detection has now made it possible to record even the rather weak SAXS signal at the level of single organelles in biological cells, as was first demonstrated in 2012, both for bacteria [159] and eukaryotic cells [160]. Scanning the sample through a micro- or nano-focused x-ray beam, the total SAXS signal around the beam stop provides an x-ray darkfield contrast for each pixel, which is sensitive to molecular structures by ways of probing Fourier components of the electron density variations. Apart from the total scattering cross-section encoded in this darkfield SAXS signal, the diffraction pattern in each image pixel can be analyzed in view of anisotropy, form factors and structure factors, see figure 24.

#### Current and future challenges.

The combination of fluorescence microscopy and SAXS, carried out on the same cell in a correlative approach, offers a powerful new approach to gain additional insight into the nanostructure of biological samples, by including structures which cannot be readily identified by fluorescence markers. Image analysis of both modalities will require automated image registry and advanced image processing. A first correlative approach was demonstrated by Weinhausen *et al* [160], recording fluorescence and x-ray darkfield signals from the same keratin filament bundles in keratin over-expressing human adrenal cortex carcinoma-derived cells.

**Figure 25.**
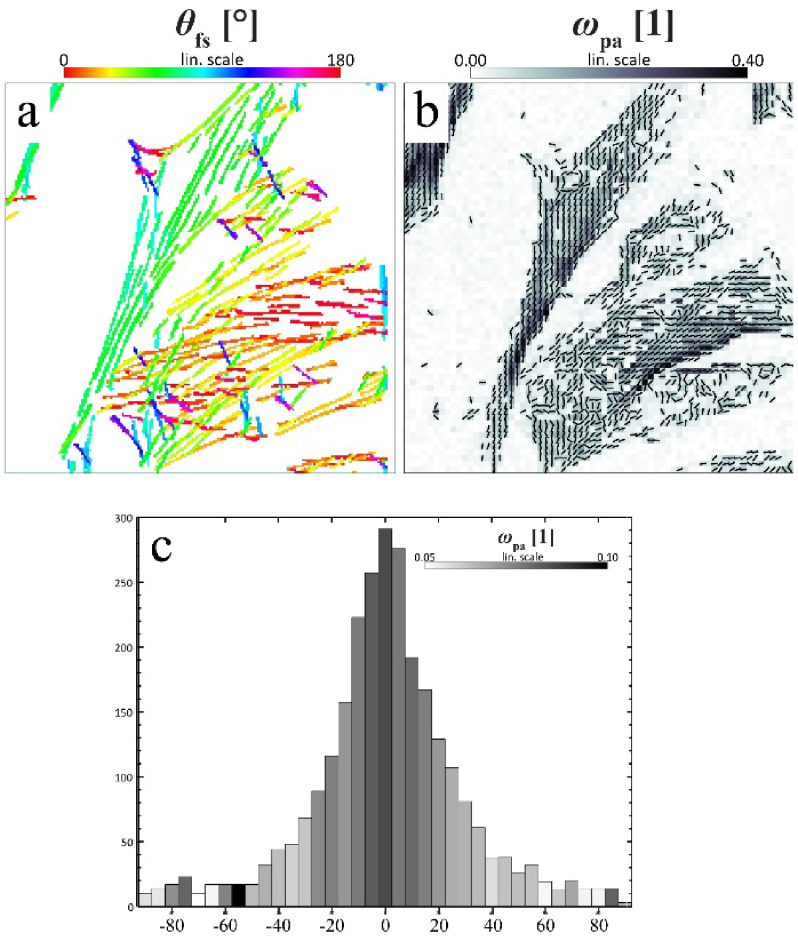
Correlating fluorescence and x-ray scattering anisotropy. (a) Most significant filaments determined by the filament sensor from a fluorescence micrograph of neonatal rat cardiac tissue. (b) PCA-results of a nano-diffraction scan showing the order parameter Ω_pa_ (grey scale) and filament orientation *θ*_pa_. (c) Histogram of the angular deviation between both methods revealing excellent correlation. Reproduced from [163]. © 2017 IOP Publishing Ltd and Deutsche Physikalische Gesellschaft. CC BY 3.0.

A subsequent study by Bernhardt *et al* [161] studied human mesenchymal stem cells, and established an automated principal component analysis (PCA) of the diffraction patterns. This allowed for the identification of the orientation of filamentous actin, as well as the degree of anisotropy, in which the actin inflicts on all scattering components in its surroundings. This approach was extended to a full correlation of acto-myosin fiber structures in cardiomyocytes by fluorescence microscopy and scanning SAXS [158]. Figure 25 shows the correlation of fluorescently labeled and identified stress fibers (top left) using the filament sensor [162] and the PCA determined orientation of the SAXS signal (top right), whereas on the bottom, the histogram of angular deviations between the two methods is shown to center around 0° [163].

Nowadays, correlation of fluorescence and SAXS signals can be readily achieved at a sufficient signal-to-noise-ratio for freeze-dried and vitrified samples, as shown in [159, 160]. However, it is highly desirable to conduct such measurements in aqueous environments and ultimately probe living cells. Towards this goal, the first SAXS recordings of living cells have been demonstrated based on SAXS compatible cell chambers in [164], and subsequently in [165]. Importantly, significant changes in the structure factor, due to chemical fixation, were demonstrated in [165]. The main challenge in live cell SAXS recordings is without a doubt the narrow window of allowable dose, assuring on one hand sufficient signal—which is more difficult in the hydrated than in freeze-dried state—and on the other hand preventing radiation damage, which is also accentuated in the living state and aqueous medium, due to the transport of free radicals.

#### Advances in science and technology to meet challenges.

Achieving SAXS for hydrated biological cells, with a signal quality that allows for quantitative analysis beyond recording a simple darkfield, is extremely challenging. It will require an optimization strategy to reach the required signal-to-noise without (or with tolerable) radiation damage. The x-ray photon energy has to be decreased with respect to the discussed prior work [159–165] probably to a range between 4–6 keV, which is largely sufficient to penetrate a cell, and which offers an increased SAXS signal per dose [166]. This, in turn, requires improvements in sample chamber design to keep the water layer and window materials sufficiently thin, and therefore the background low. Here, windows of crystalline freestanding thin films are best suited. Microfluidic exchange of the (degassed) solution is required as well as high quantum efficiency in detection, and careful cleaning of the beam from spurious SAXS background. Moreover, spatial resolution has to be sacrificed. Sufficient SAXS quality may be reached only for spot sizes in the micron range, but not in the nano focus range. All of these requirements are useful but not indispensable for cryogenic (vitrified) cells. Here, the exploitable dose window is extended to 10^8^ Gy. The challenge here is sample preparation and transfer to a cryo-compatible scanning stage, which is not commercially available. Contrarily, freeze-dried cells will be relatively easy to record, but of course only of limited interest.

Ultimately, one also wants to combine super-resolution light microscopy *in situ* with scanning x-ray scattering methods. However, this challenge has certain technical limits, e.g. no current immersion optics are applicable, as the sample needs to be kept in air for x-ray measurements. A novel setup allowing STED microscopy, with an air objective in a horizontal geometry directly at a nano focus synchrotron beamline suitable for scanning SAXS, is under way (Bernhardt *et al*, unpublished). This will also be extremely useful to shed light on the mechanisms and constraints of radiation damage, since the cells can be monitored by STED directly after x-ray exposure. Finally, novel analysis schemes are required to quantify structural information recorded in a combination of real space (by scanning), as well as in reciprocal space (by recording of diffraction patterns). Importantly, the real space data can also help to guide the formulation of suitable models to interpret the scattering signal.

#### Concluding remarks.

We briefly reviewed the current status of correlating fluorescence microscopy and scanning x-ray scattering on biological cells and laid out some ideas for the future. In particular, the combination of scanning SAXS with STED microscopy is promising to shed light on cellular components, such as the nano structure of the cytoskeleton, the nuclear envelope, and DNA organization. In the future, correlative approaches may help to elucidate how those structures are involved in the complex process of transcription and gene regulation. Probing the unlabeled local electron density, in addition to recording labeled biomolecules, will bring the up to now invisible structures ‘to light’.
